# Supplementary material for: Retrofusion of intralumenal MVB membranes parallels viral infection and coexists with exosome release
Source: Curr Biol. 2021 Sep 13;31(17):3884–3893.e4. doi: 10.1016/j.cub.2021.06.022 (PMC8445322; doi:10.1016/j.cub.2021.06.022)
Supplement: Document S1. Figures S1–S4 [file mmc1.pdf]

Current Biology, Volume *31*

Supplemental Information

Retrofusion of intralumenal MVB membranes parallels viral infection and coexists with exosome release

Priscillia Perrin, Lennert Janssen, Hans Janssen, Bram van den Broek, Lennard M. Voortman, Daphne van Elsland, Ilana Berlin, and Jacques Neefjes

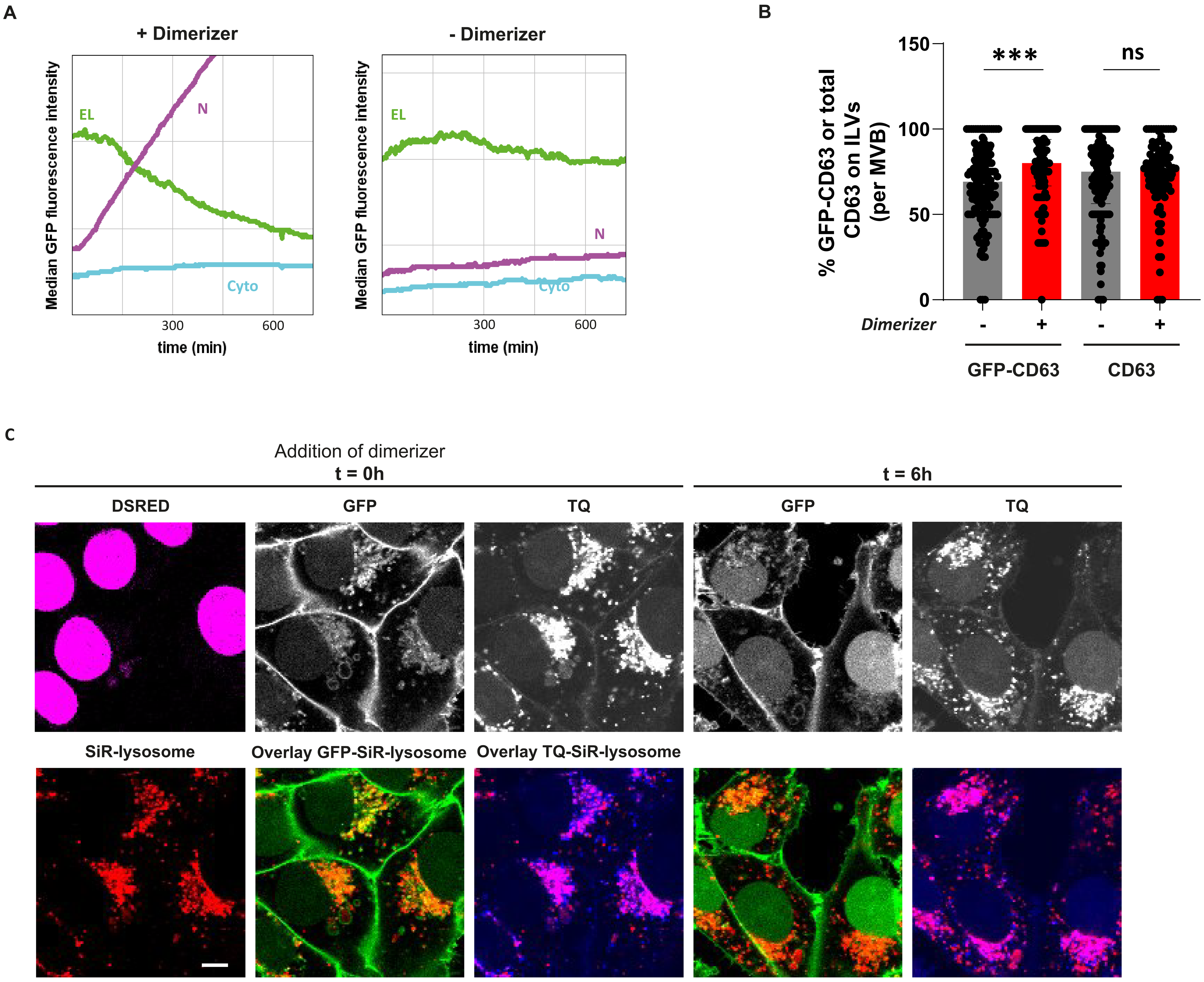
A
+ Dimerizer
- Dimerizer
Median GFP fluorescence intensity
EL
N
Cyto
300
600
time (min)
B
% GFP-CD63 or total CD63 on ILVs (per MVB)
150
100
50
0
***
ns
Dimerizer
-
+
GFP-CD63
CD63
C
Addition of dimerizer
t = 0h
t = 6h
DSRED
GFP
TQ
SiR-lysosome
Overlay GFP-SiR-lysosome
Overlay TQ-SiR-lysosome

Figure S1. Analysis of retrofusion measurement and TEV protease efficiency. Related to Figures 1 and 2.

(A) Representative plot of intracellular median GFP fluorescence intensity from GFP-CD63 cells either (+) or not (-) cultured with dimerizer for the time indicated (min) N: nucleus; EL late endosomes as labelled by SiR-lysosome; Cyto: cytoplasm. EL: endolysosome; N: nucleus; PM: plasma membrane; Cyto: cytoplasm.

(B) Quantification of GFP-CD63 as detected by immunogold labeling on ILVs relative to LM (expressed as ratio per MVB) following incubation with dimerizer (6h). Shown is median +/- IQR from over 98 MVBs from 2 independent experiments.

(C) Analysis of Lamp1-TEV-TQ expression in GFP-CD63 retrofusion-monitoring cells stained with SiR-lysosome. Confocal fluorescence images of GFP-CD63 and Lamp1-TEV-TQ (*white*) distribution before (t=0h) and after (t=5h) treatment with dimerizer are shown, along with color overlays of GFP (*green*) or TQ (blue) with SiR-Lysosome (*red*). NLS-DsRED (*magenta*) in the nucleus indicate expression of the split TEV protease at t=0. Bar: 10 µm. TQ : mTurquoise2.

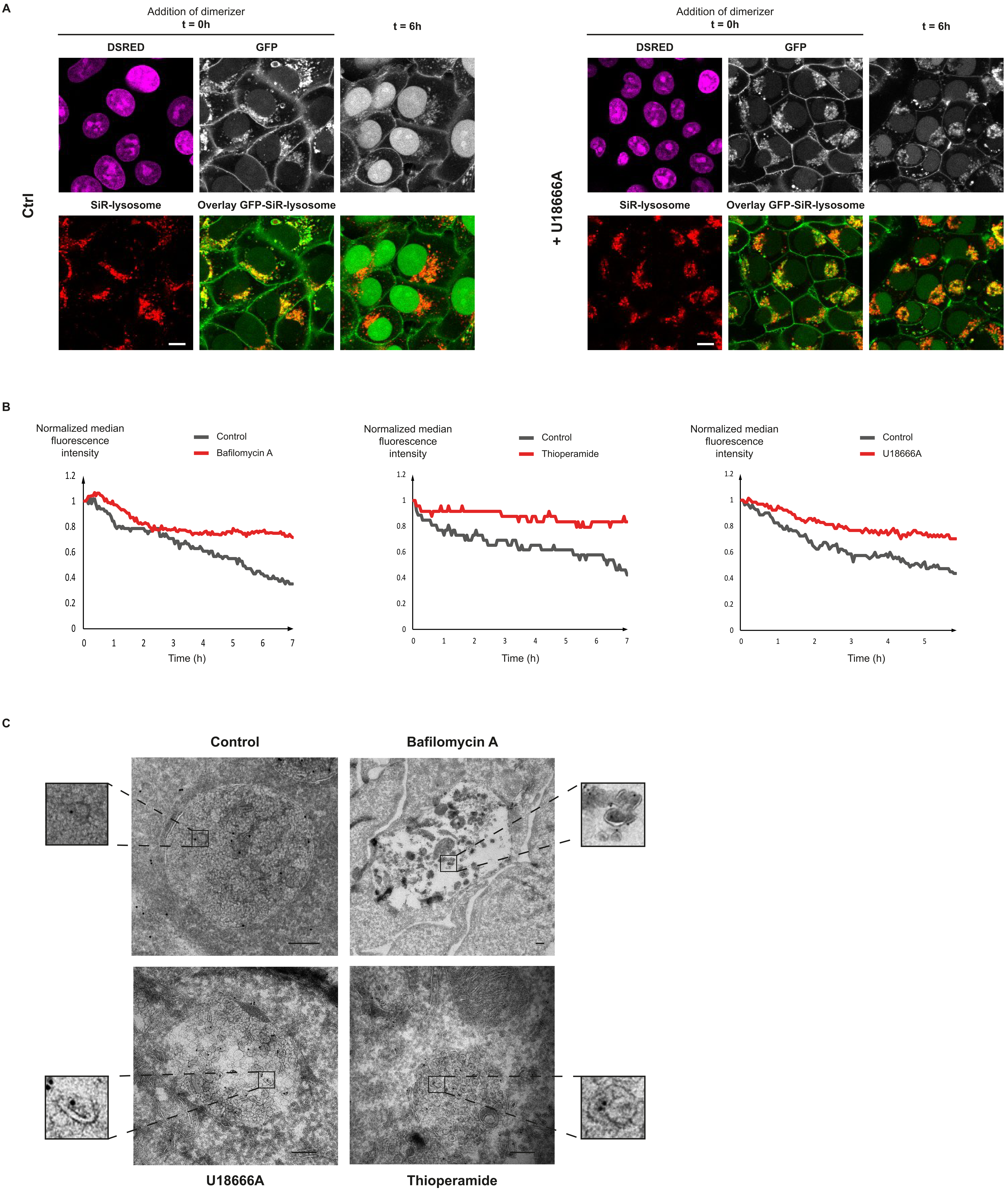
A
Addition of dimerizer
t = 0h
t = 6h
DSRED
GFP
Ctrl
SiR-lysosome
Overlay GFP-SiR-lysosome
+ U18666A
B
Normalized median fluorescence intensity
Control
Bafilomycin A
Thioperamide
U18666A
1.2
1
0.8
0.6
0.4
0.2
0
0 1 2 3 4 5 6 7
Time (h)
C
Control
Bafilomycin A
U18666A
Thioperamide

Figure S2. Effects of lipid and pH manipulation on the rate of ILV retrofusion. Related to Figure 3.

(A) Confocal images of control (left panel; Ctrl) and U18666A treated (right panel) GFP-CD63 cells as used for monitoring retrofusion. Confocal fluorescence images of GFP-CD63 (*white*) distribution before (t=0h) and after (t=6h) treatment with dimerizer are shown, along with color overlays of GFP (*green*) with SiR-Lysosome (*red*). NLS-DsRED (*magenta*) in the nucleus indicate expression of the split TEV protease at t=0. All scale bars, 10 µm.

(B) Representative plots of normalized median GFP fluorescence intensity in endolysosomes (as stained with SiR-lysosome) over time (min) following dimerizer addition in control cells (*gray*) versus those treated with Bafilomycin A, thioperamide or U18666A (*red*).

(C) Electron micrograph featuring immunogold labeling with GFP antibody of control cells following incubation with U18666A, thioperamide or Bafilomycin A. Scale bar, 200 nm.

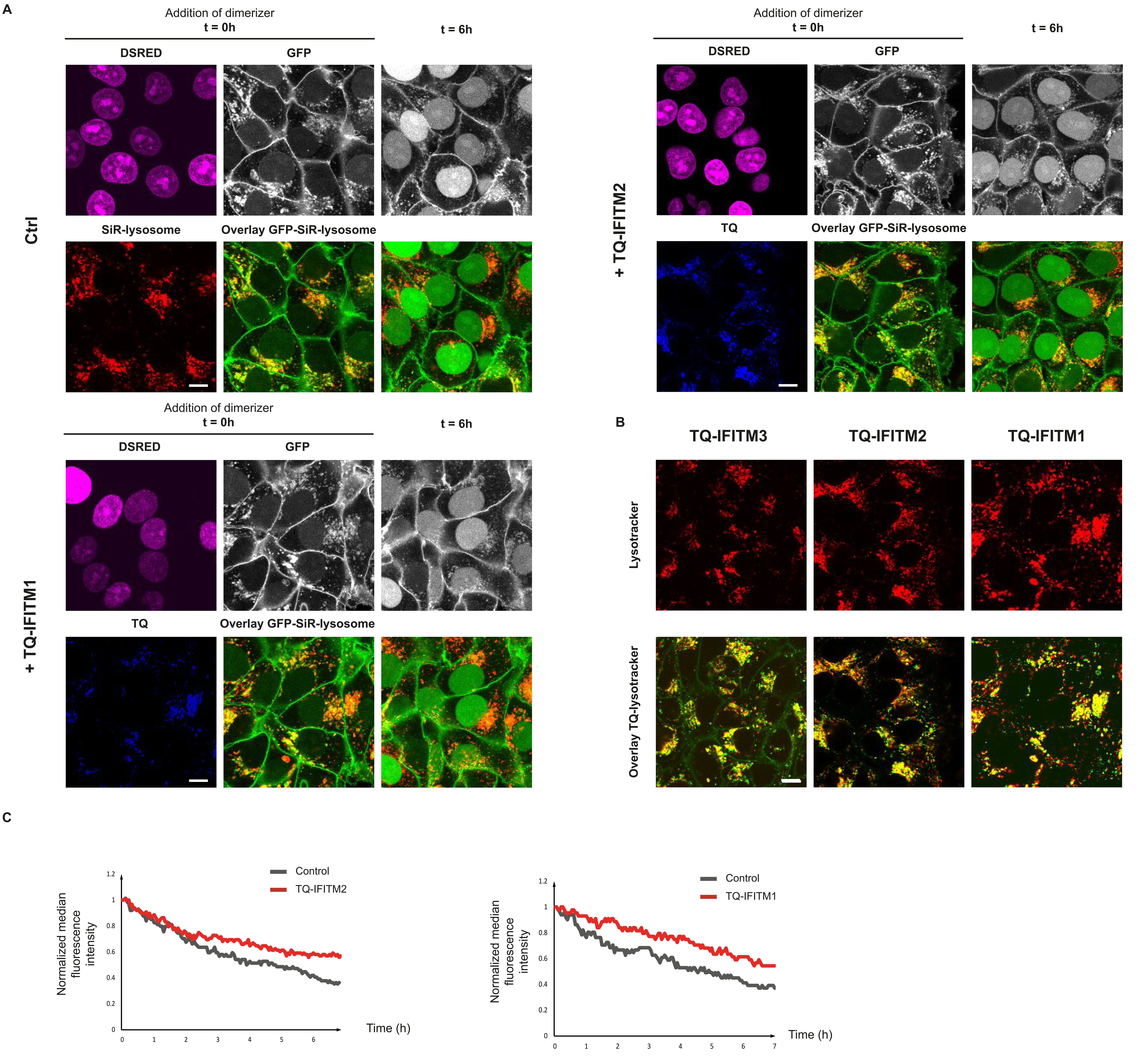
A
Addition of dimerizer
t = 0h
t = 6h
DSRED
GFP
Ctrl
SiR-lysosome
Overlay GFP-SiR-lysosome
+ TQ-IFITM2
TQ
+ TQ-IFITM1
B
TQ-IFITM3
TQ-IFITM2
TQ-IFITM1
Lysotracker
Overlay TQ-lysotracker
C
Normalized median fluorescence intensity
Control
TQ-IFITM2
TQ-IFITM1
Time (h)

Figure S3. Effects of overexpression of the IFITM family members on the rate of ILV retrofusion. Related to Figure 4.

(A) Confocal images showing overexpression of IFITM1 or 2 in GFP-CD63 retrofusion-monitoring cells, as used for the retrofusion experiments. Confocal fluorescence images of GFP-CD63 (*white*) distribution before (t=0h) and after (t=6h) treatment with dimerizer are shown, along with color overlays of GFP (*green*) with SiR-Lysosome (*red*). NLS-DsRED in the nucleus (*magenta*) and TQ-IFITM1 and 2 (*blue*) respectively indicate expression of the split TEV protease and the IFITM proteins at t=0. Scale bars, 10 µm

(B) Localization of TQ-IFITM1, 2 and 3 proteins to late endocytic compartments as marked by SiR-Lysosome. Confocal fluorescence overlays of TQ-IFITM1-3 (*green*) with SiR-Lysosome (*red*) are shown in the bottom panel.

(C) Representative plots of normalized median GFP fluorescence intensity in endolysosomes (as marked by SiR-Lysosomes) over time (min) following dimerizer addition in control cells (*gray*) versus those overexpressing TQ-IFITM1 or 2 (*red*).

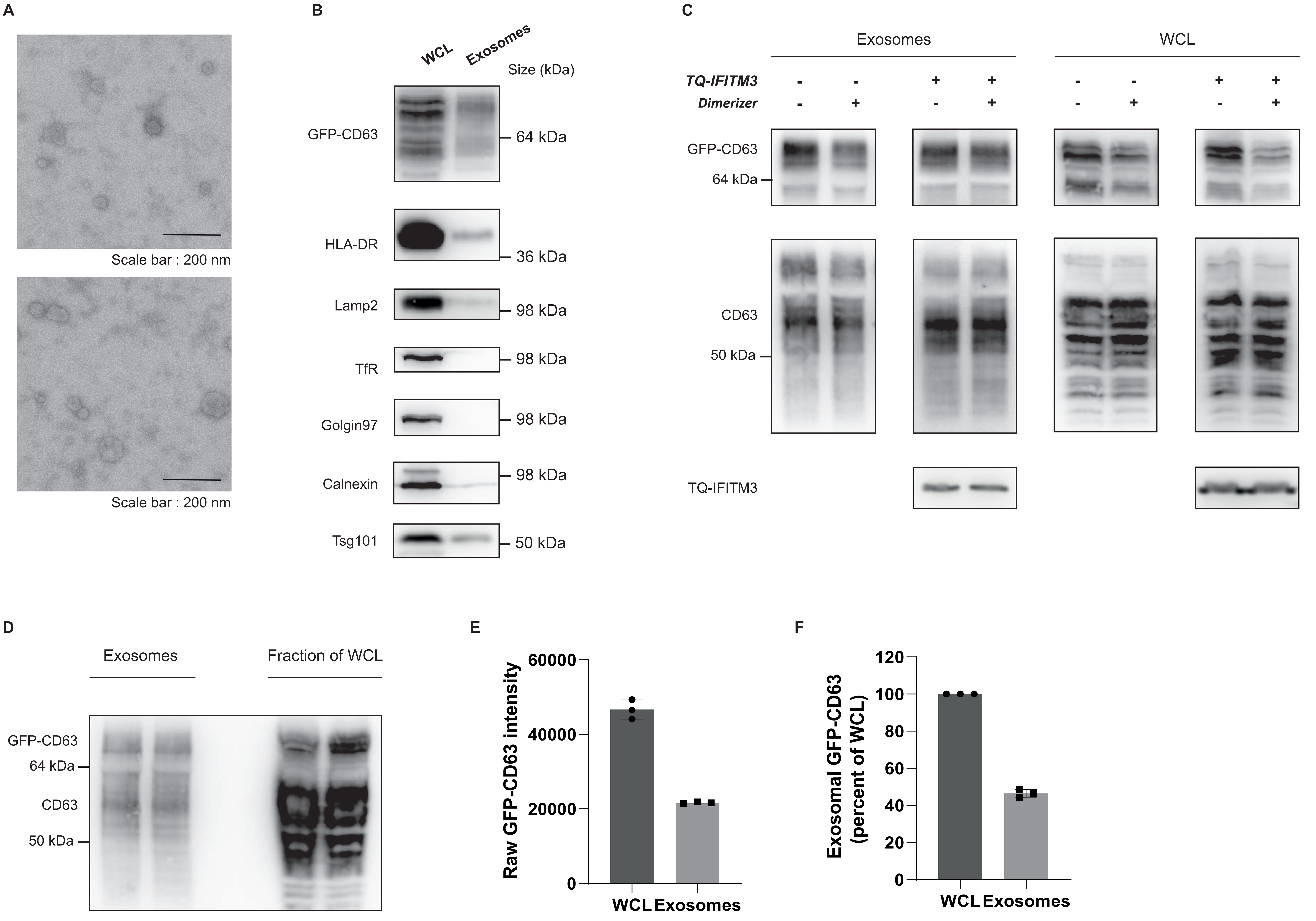

G

GFP-CD63	*WCL*	*MVBs*
Degradation + secretion	11%	18%
Degradation	5%	8%
Secretion	6%	10%

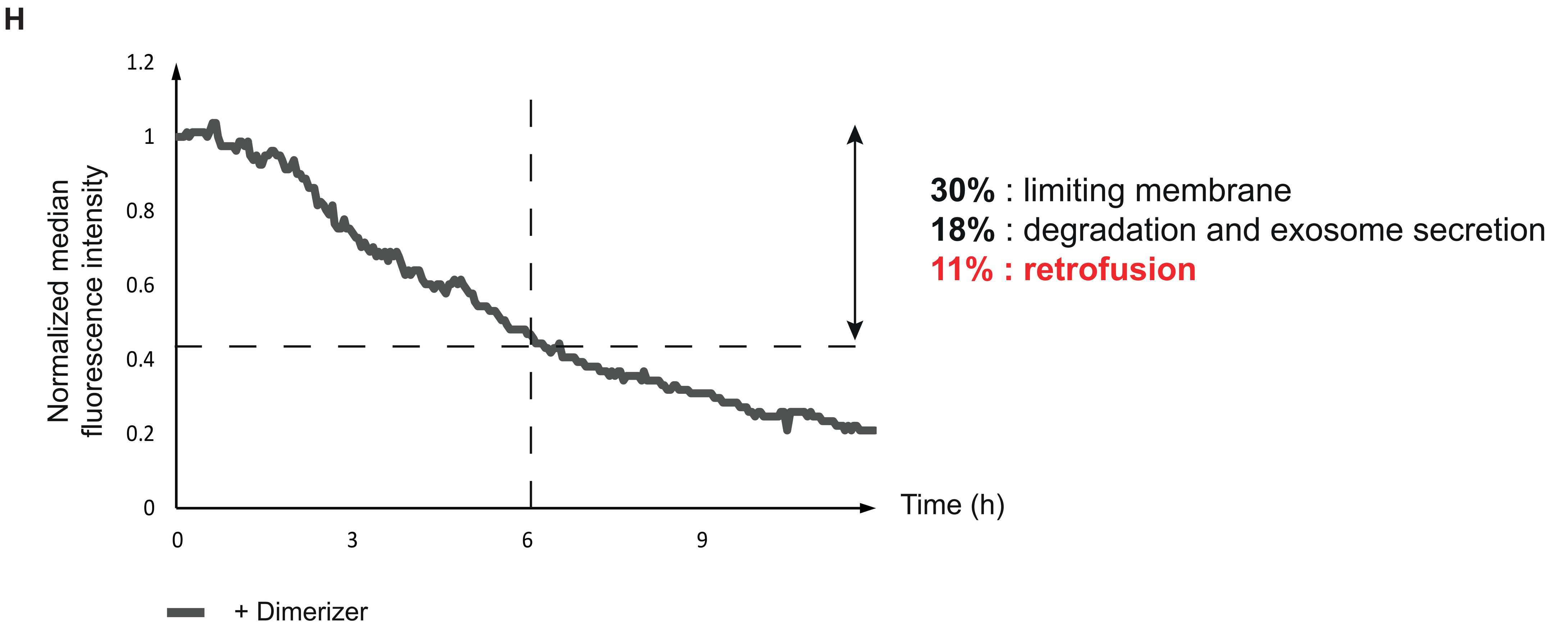

Figure S4. Retrofusion and exosomes. Related to Figure 4.

(A) Electron micrographs of exosome isolates prepared as described in Figure 4F. Scale bar, 200 nm.

(B) Biochemical profile of exosome isolates (secreted in absence of dimerizer) was assessed by immunoblot against the indicated markers. TfR: transferrin receptor, recycling endosomes; HLA-DR: MHC class II, LM and ILVs of late endosomes/lysosomes; Lamp2: LM of late endosomes/lysosomes; Golgin97: trans-Golgi network; Calnexin: endoplasmic reticulum; Tsg101: exosome marker. WCL: diluted whole-cell lysate (to prevent overloading control signal). The position of marker proteins is indicated.

(C) Immunoblot analysis of GFP-CD63 (detected with anti-GFP antibodies) versus untagged CD63 in exosome isolates and a fraction of whole cell lysate (WCL) from control cells or cells overexpressing TQ-IFITM3 treated in the presence (+) or absence (-) of dimerizer. The different incubations as well as the position of the marker proteins are indicated.

(D) Immunoblot analysis of CD63 in exosome isolates and WCL from control cells. Exosomes were isolated from $10x10^6$ cells. For the WCL, $1.3x10^6$ cells were loaded on the same gel. The results from a duplicate exosome isolation were loaded on two lanes as is the corresponding WCL. Position of marker proteins is indicated.

(E) Quantification of GFP-CD63 signal in exosome isolates versus the signal in a fraction of WCL from control cells. Exosomes were isolated from $10x10^6$ cells and $1.3x10^6$ cells were loaded on the same gel. Shown is mean +/- SD from three independent experiments.

(F) Quantification of the percentage of GFP-CD63 secreted in exosomes relative to the fraction cell lysate ($1.3x10^6$ cells) including MVBs. This is not corrected for the number of cells providing exosomes. The corrected values are shown in Figure 4H.
