# Supplementary material for: Retrofusion of intralumenal MVB membranes parallels viral infection and coexists with exosome release
Source: Curr Biol. 2021 Sep 13;31(17):3884–3893.e4. doi: 10.1016/j.cub.2021.06.022 (PMC8445322; doi:10.1016/j.cub.2021.06.022)
Supplement: Document S2. Article plus supplemental information [file mmc6.pdf]

Report

Current Biology

Retrofusion of intralumenal MVB membranes parallels viral infection and coexists with exosome release

Graphical abstract

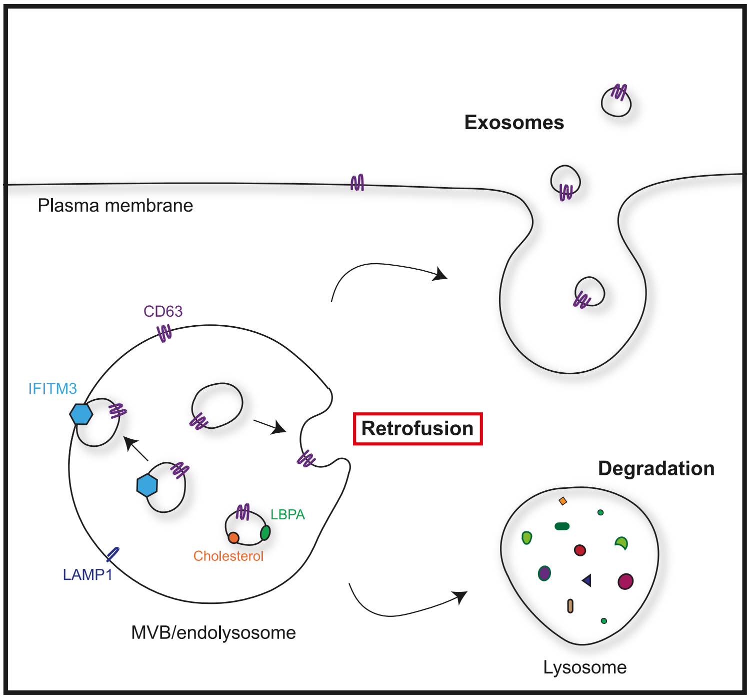

Authors

Priscillia Perrin, Lennert Janssen, Hans Janssen, ..., Daphne van Elsland, Ilana Berlin, Jacques Neefjes

Correspondence

j.j.c.neefjes@lumc.nl

In brief

Multivesicular bodies (MVBs) are complex organelles harboring internal vesicles. Using a chemically controlled system, Perrin et al. visualize a part of these internal vesicles fusing back to the limiting membrane. This process of retrofusion exists in equilibrium with lysosomal degradation and exosome release and is inhibited by antiviral proteins.

Highlights

- MVBs are complex organelles with intraluminal vesicles bound by the limiting membrane
- Intraluminal membranes are in a dynamic equilibrium with the limiting membrane
- Retrofusion of internal vesicles is controlled by processes used for viral fusion
- Exosomes arise from internal MVB vesicles not participating in retrofusion

Perrin et al., 2021, Current Biology *31*, 3884–3893
September 13, 2021 © 2021 The Author(s). Published by Elsevier Inc.
https://doi.org/10.1016/j.cub.2021.06.022

CellPress
OPEN ACCESS

Current Biology

Report

Retrofusion of intralumenal MVB membranes parallels viral infection and coexists with exosome release

Priscillia Perrin,[1] Lennert Janssen,[1] Hans Janssen,[2] Bram van den Broek,[3,4] Lennard M. Voortman,[1] Daphne van Elsland,[1] Ilana Berlin,[1] and Jacques Neefjes[1,5,*]

[1]Oncode Institute, Department of Cell and Chemical Biology, Leiden University Medical Center, Einthovenweg 20, 2333 ZC Leiden, the Netherlands
[2]Division of Biochemistry, Netherlands Cancer Institute, Plesmanlaan 121, 1066 CX Amsterdam, the Netherlands
[3]BioImaging Facility, Netherlands Cancer Institute, Plesmanlaan 121, 1066 CX Amsterdam, the Netherlands
[4]Division of Cell Biology, Netherlands Cancer Institute, Plesmanlaan 121, 1066 CX Amsterdam, the Netherlands
[5]Lead contact
*Correspondence: j.j.c.neefjes@lumc.nl
https://doi.org/10.1016/j.cub.2021.06.022

SUMMARY

The endosomal system constitutes a highly dynamic vesicle network used to relay materials and signals between the cell and its environment.[1] Once internalized, endosomes gradually mature into late acidic compartments and acquire a multivesicular body (MVB) organization through invagination of the limiting membrane (LM) to form intraluminal vesicles (ILVs).[2] Cargoes sequestered into ILVs can either be delivered to lysosomes for degradation or secreted following fusion of the MVB with the plasma membrane.[3] It has been speculated that commitment to ILVs is not a terminal event, and that a return pathway exists, allowing "back-fusion" or "retrofusion" of intraluminal membranes to the LM.[4] The existence of retrofusion as a way to support membrane equilibrium within the MVB has been widely speculated in various cell biological contexts, including exosome uptake[5] and major histocompatibility complex class II (MHC class II) antigen presentation.[6–9] Given the small physical scale, retrofusion of ILVs cannot be measured with conventional techniques. To circumvent this, we designed a chemically tunable cell-based system to monitor retrofusion in real time. Using this system, we demonstrate that retrofusion occurs as part of the natural MVB lifestyle, with attributes parallel to those of viral infection. Furthermore, we find that retrofusion and exocytosis coexist in an equilibrium, implying that ILVs inert to retrofusion comprise a significant fraction of exosomes destined for secretion. MVBs thus contain three types of ILVs: those committed to lysosomal degradation, those retrofusing ILVs, and those subject to secretion in the form of exosomes.

RESULTS AND DISCUSSION

To address the challenge of observing retrofusion in real time, we designed a chemically tunable reporter system to visualize and monitor the occurrence of this process in living cells. To follow relocalization of membrane components from the intraluminal vesicles (ILVs) back to the limiting membrane (LM), we furnished the canonical multivesicular body (MVB) marker tetraspanin membrane protein CD63 with a GFP tag harboring an N-terminal nuclear localization signal (NLS) and a C-terminal tobacco etch virus (TEV) protease-specific cleavage site (TCS), resulting in NLS-GFP-TCS-CD63 stably expressed in MelJuSo cells, henceforth referred to as GFP-CD63 (Figures 1A and 1B). The TEV protease was introduced into the same cells as two inactive parts, coexpressed along with NLS-DsRED from a single polycistronic vector (Figures 1A and 1B), which can be brought together on demand by a rapamycin analog termed "dimerizer."[10] Upon addition of dimerizer, protease activity would be reconstituted and result in cleavage of available GFP-CD63 (i.e., GFP exposed to the cytosol, unlike the GFP in ILVs). As a consequence, CD63 at the LM of MVBs would lose its GFP fluorescence, and newly liberated NLS-GFP would be targeted to the nucleus, averting accumulation of cytosolic background (Figures 1A and 1B).

In the absence of dimerizer, GFP-CD63 localized to both ILVs and the LM of MVBs at a 2:1 ratio (Figures 1C and 1D), mirroring the distribution of endogenous CD63,[11] whereas the split protease remained inactive (Figure 1B), and no nuclear GFP was observed (Figure 2A; t = 0). GFP-CD63 was also present at the cell surface (Figure 2A). Upon addition of dimerizer to the cells, and consequent reconstitution of the TEV protease (Figure 1B), GFP fluorescence in acidic vesicles of the endocytic tract marked by SiR-lysosome began to diminish over time, accompanied by a concomitant rise of GFP signal in the nucleus (Figure 2A; Video S1). Because GFP in the nucleus can arise from cleaved CD63 molecules in endosomes as well as from the cell surface, we directly measured the decay of GFP fluorescence in the late endosomes. To correct for background fluorescence, cytoplasmic GFP was subtracted from the SiR-lysosome-positive GFP signal,

 © 2021 The Author(s). Published by Elsevier Inc.
This is an open access article under the CC BY license (http://creativecommons.org/licenses/by/4.0/).

CellPress
OPEN ACCESS

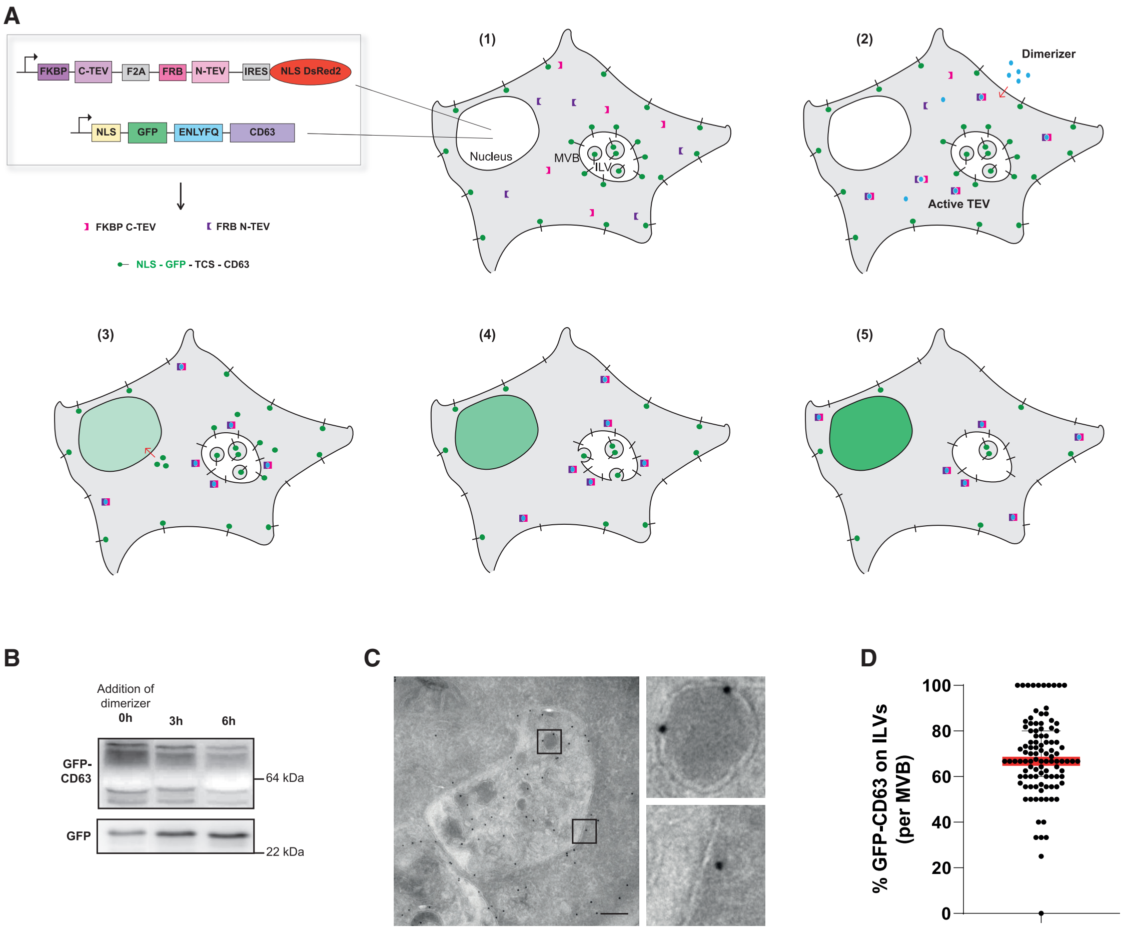

Figure 1. Design of a cell-based reporter system to monitor retrofusion

(A) Concept of the chemically controlled system for visualizing retrofusion. (1) MelJuSo cells coexpress NLS-GFP-TCS-CD63 (GFP-CD63; green), localized on the LM and ILVs of multivesicular ELs and on the plasma membrane, and cytoplasmic TEV protease fragments FRB-N-TEV (pink) and FKBP-C-TEV (purple) co-translated with nuclear NLS-DsRED2. In the absence of dimerizer (–), the split TEV protease is inactive. (2) Dimerizer (blue) addition (+) reconstitutes TEV protease activity. (3) Active TEV protease cleaves NLS-GFP from GFP-CD63 as it is exposed to the cytoplasm. (4) Upon ILV retrofusion, additional GFP-CD63 becomes a substrate for the TEV protease. (5) Time-dependent loss of GFP signal from the MVB (as labeled by SiR-lysosome), accompanied by a rise in nuclear GFP, is monitored and quantified. NLS, nuclear localization signal; TCS, TEV cleavage site; EL, endolysosome; LM, limiting membrane; ILV, intralumenal vesicle.

(B) Immunoblot analysis of dimerizer-induced TEV protease reconstitution and GFP-CD63 cleavage over time. Whole-cell lysates (WCLs) were immunostained with GFP antibody; the top and bottom bands show GFP-CD63 and free GFP, respectively. The position of marker proteins is indicated.

(C) Electron micrograph featuring immunogold labeling with GFP antibody of GFP-CD63 control cells. Zoomed insets show some 10-nm gold particles on ILVs and the LM. Scale bar, 200 nm.

(D) Quantification of GFP-CD63 abundance on ILVs relative to the LM (expressed as the ratio per MVB) as assessed by immunogold labeling. Data were collected from 64 MVBs from n = 2 independent experiments. Shown is median ± interquartile range (IQR).

yielding normalized fluorescence intensity associated with endolysosomal organelles over time (Figures 2B and S1A; Video S2). Following the initial equilibration phase (Figures 2B and S1A; t = 0–90 min), a near-linear decay of GFP signal from SiR-lysosome-positive compartments was observed (t = 90–360 min) and its slope A was assessed (Figures 2A, 2B, and 2C; Video S1). This decay was contingent on TEV protease activity, as evidenced by no appreciable loss of endolysosomal GFP (or rise in nuclear GFP) in the absence of dimerizer treatment (Figures 2A, 2B, and 2C; Video S3).

Because only GFP exposed on the cytosolic side of the LM can be accessible for TEV cleavage, we expected that, in the absence of retrofusion, dimerizer-induced decay of endolysosomal GFP fluorescence would plateau at roughly 66%–70%, corresponding to the amount of intraluminal GFP-CD63 (Figure 1D). Further loss of this GFP signal would necessitate repopulation of the LM by GFP-CD63 derived from retrofusing ILVs. Inspection of the GFP signal decay following the system's engagement with dimerizer (Figures 2B and S1A) revealed that 41% ± 5% of the starting GFP signal associated with endolysosomes remained at 6 h post treatment (Figures 2B and 2D), implying that a proportion of GFP signal lost from endocytic compartments depends on the occurrence of retrofusion. Indeed, electron microscopy (EM) analysis following 6-h incubation with dimerizer and immunogold labeling against both GFP and CD63 demonstrated a decrease in GFP staining relative

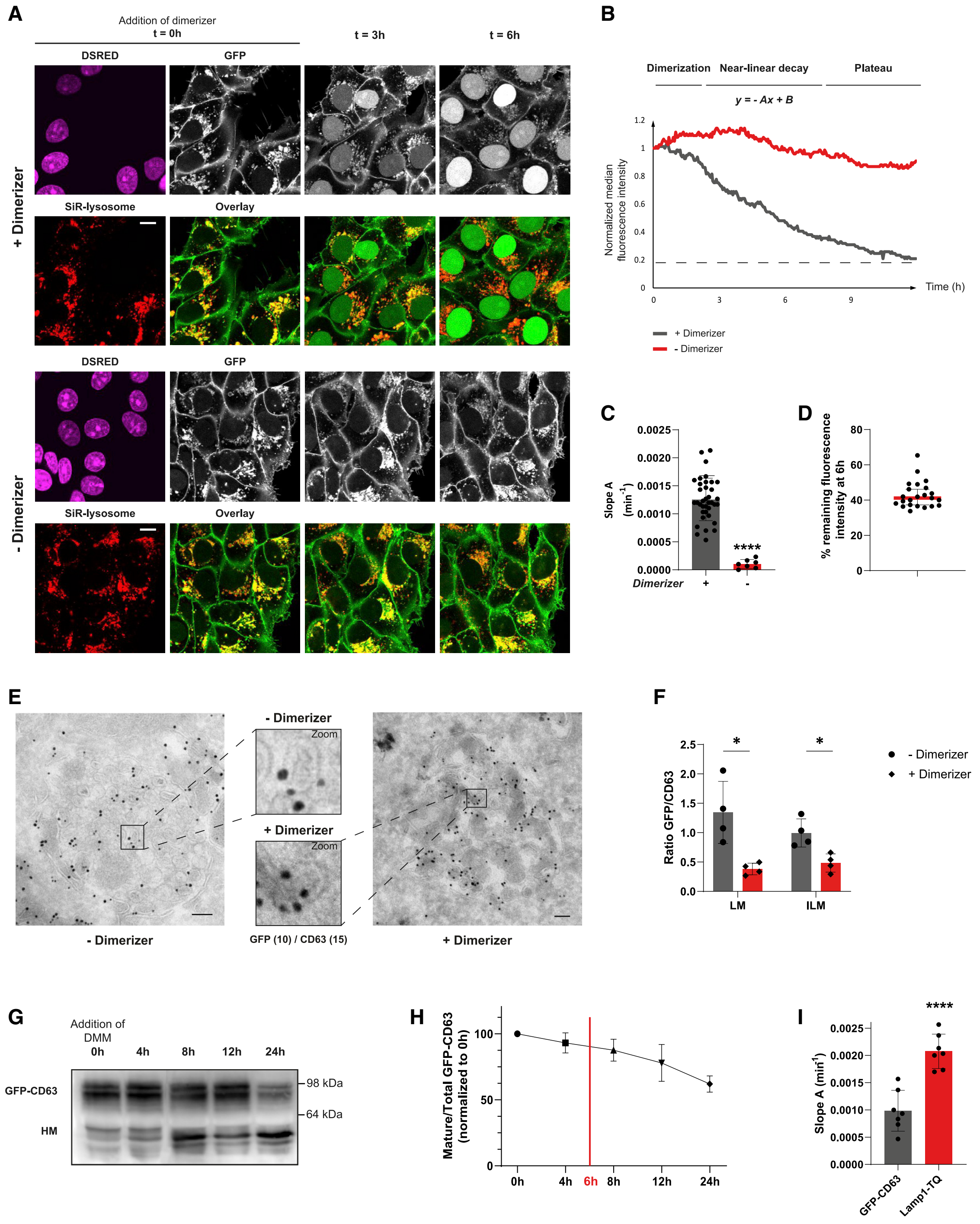

Figure 2. Monitoring retrofusion of GFP-CD63-positive ILVs in real time

(A) Representative confocal fluorescence stills from a time-lapse experiment with GFP-CD63 cells (white) imaged in the presence (+) or absence (–) of dimerizer along with color overlays of GFP (green) with SiR-lysosome (red) marking ELs are shown. DsRED-positive (magenta) nuclei indicate expression of the split TEV protease at t = 0. Scale bars, 10 μm.

(B) Representative plot of EL-associated median GFP fluorescence intensity over time (min), corrected by cytoplasmic GFP background subtraction; (+), dimerizer (gray); (–), dimerizer (red) treatment. Slope A characterizes the near-linear GFP decay. Only loss of GFP signal in MVBs is quantified in our experiments as nuclear NLS-GFP is also provided by GFP-CD63 molecules at the cell surface.

(C) Quantification of slope A relative to (+) dimerizer. Shown is mean ± SD from n = 7 independent experiments for (–) dimerizer and n = 35 for (+) dimerizer.

(D) Quantification of GFP fluorescence remaining in late endosomes at 6 h after dimerizer addition, expressed as % GFP fluorescence at t = 0. Shown is mean ± SD from n = 23 independent experiments.

(legend continued on next page)

to that of CD63 on both the LM and intraluminal membranes (ILM) (Figures 2E and 2F), confirming intraluminal GFP recovery. Of note, dimerizer treatment did not affect the distribution of CD63 (ILM versus LM) in MVBs (Figure S1B). Past the 6-h time point, the decay of endolysosomal GFP began to plateau (Figures 2B and S1A) and, by 9 h post dimerizer addition, 20% of the initial fluorescence intensity still remained in MVBs. This suggests that a significant fraction of GFP-CD63-positive ILVs does not participate in retrofusion and is likely subject to other pathways.

Because MVBs continuously receive input of new membranes and sort cargoes toward diverse fates, notably including lysosomal proteolysis, we tested whether biosynthesis or degradation of GFP-CD63 contributes to the GFP decay measured during the time frame of our microscopy assay. A pulse-chase experiment with 1-deoxymannosidase I (DMM), an inhibitor of mannosidase I,[12] was used to assess the relative proportion of old GFP-CD63 bearing long polylactosaminoglycans versus newly synthesized GFP-CD63 carrying high-mannose N-linked glycans (migrating at a lower molecular weight on the gel) over time (Figure 2G), yielding a half-life longer than 24 h (Figure 2H), which was expected because CD63 is known to be highly stable.[13] These data indicate that the natural turnover of GFP-CD63 does not significantly contribute to the decay of endolysosomal GFP fluorescence following dimerizer addition.

Because the readout of our system is contingent on the performance of the TEV protease, we examined whether the decay of GFP-CD63 is limited by the rate of TEV-mediated cleavage. To this end, the cytoplasmic tail of the lysosomal membrane protein LAMP1, which localizes almost exclusively to the LM of MVBs,[14] was fused to a TEV cleavage site followed by mTurquoise2-NLS, and the resulting LAMP1-TEV-TQ (turquoise) construct was stably coexpressed with the TEV protease and GFP-CD63. Following addition of dimerizer, the mTurquoise2 signal associated with MVBs decreased more rapidly and reached a lower end value than its GFP-CD63 counterpart expressed in the same cells (Figures 2I and S1C), indicating that inaccessibility of ILV-localized GFP-CD63 to the TEV protease, rather than TEV cleavage efficacy, is the limiting factor in our measurements.

MVBs are acidic organelles, which is fundamental to their proteolytic function and also, intriguingly, crucial to fusion of many viruses with the LM.[15] To test whether retrofusion of ILVs also benefits from the acidic environment of the MVB, endolysosomal pH was elevated using bafilomycin A, a V-ATPase inhibitor preventing endosomal acidification[16] (Figure 3A), and retrofusion of GFP-CD63 was monitored as before. Following addition of dimerizer, the GFP fluorescence decay quickly reached a plateau, with 83% of the initial fluorescence intensity remaining in MVBs (Figures 3B, 3C, and S2B), suggesting that only GFP-CD63 exposed at the LM was cleaved off by the TEV protease. EM analysis confirmed that the relative distribution of GFP-CD63 between the LM and ILVs of MVBs in the presence of bafilomycin A was similar to that observed under control conditions (Figures 1C, 1D, 3D, and S2C) and was thus not responsible for the altered GFP decay. Thus, like other processes occurring at the MVB, constitutive retrofusion involves acidification of these organelles.

As a complex and agile cargo-sorting platform, amenable to repeated membrane deformation, the MVB carefully curates its lipid distribution between the LM and ILVs.[17] For instance, cholesterol and lysobisphosphatidic acid (LBPA) predominate on ILVs and have been speculated to modulate ILV dynamics.[18,19] We therefore tested whether altering the concentration of these lipids on ILVs could affect their propensity for retrofusion. Endolysosomal levels of LBPA or cholesterol were selectively increased using thioperamide maleate, an inverse agonist of the histamine H3 receptor HRH3,[20] or the U18666A compound, an inhibitor of lysosomal cholesterol export,[21] respectively (Figure 3A). Following dimerizer addition, the decay of GFP fluorescence from endolysosomal compartments was markedly attenuated in response to either perturbation relative to control (Figures 3B, 3C, S2A, and S2B), suggesting that accumulation of LBPA and cholesterol influences retrofusion. Once again, no significant effect on the distribution of GFP-CD63 in MVBs, nor the intraluminal content, was observed in the presence of thioperamide, implying that the resulting retrofusion attenuation cannot be attributed to lack of intraluminal GFP-CD63 (Figures 1C, 1D, 3D, 3E, and S2C). In the case of U18666A, a slight increase in intraluminal GFP-CD63 was observed, which may be attributed to attenuation of retrofusion, although lipid alterations might also affect the formation of ILVs. Based on this evidence, we propose that lipid composition of ILVs is crucial for recycling of the ILM to the LM. Because LBPA and cholesterol have both been implicated in viral infection,[22–24] these results again hint at fundamental parallels between intra-endosomal equilibrium and viral escape, explored below.

The first experimental suggestions regarding the existence of retrofusion were made, albeit indirectly, in the context of viral infection,[24] and it has been proposed that some viruses hijack the retrofusion pathway to escape lysosomal degradation and

(E) Electron micrograph featuring immunogold labeling with GFP$^{10\ nm}$ and CD63$^{15\ nm}$ gold antibodies of GFP-CD63-expressing cells in the absence or 6 h in the presence of dimerizer. Scale bars, 100 nm.

(F) Quantification of GFP-CD63 and untagged CD63 abundance on ILVs and the LM in the presence or absence of dimerizer, expressed as the ratio of GFP/CD63 (GFP and CD63, respectively, representing the sum of GFP and CD63 in all MVBs) assessed by immunogold labeling. Shown is mean ± SD from over 25 MVBs from n = 4 independent experiments.

(G) Immunoblot analysis of GFP-CD63 turnover. The mannosidase I inhibitor DMM is added to the cells at t = 0 h and cells are grown for the times indicated followed by western blot (WB) analyses and staining with GFP antibody. DMM yields CD63 molecules with high-mannose glycans (HM) that run at a lower position than mature CD63, as indicated. The position of marker proteins is indicated.

(H) Quantification of mature GFP-CD63 signal normalized to total GFP (sum of mature and HM GFP-CD63 signal) and normalized to t=0 h at each time point following DMM addition. Shown is mean ± SD from n = 4 independent experiments.

(I) Quantification of slope A of GFP-CD63 relative to TQ-Lamp1 decay following dimerizer addition. Shown is mean ± SD for n = 7 independent experiments. For primary data and cell images, see Figures S1A and S1B.

Statistical differences between the groups were assessed using unpaired Student's t test (C and I) or multiple t test (F) (*p < 0.05, **p < 0.01, ***p < 0.001, ****p < 0.0001). See also Figure S1 and Videos S1, S2, and S3.

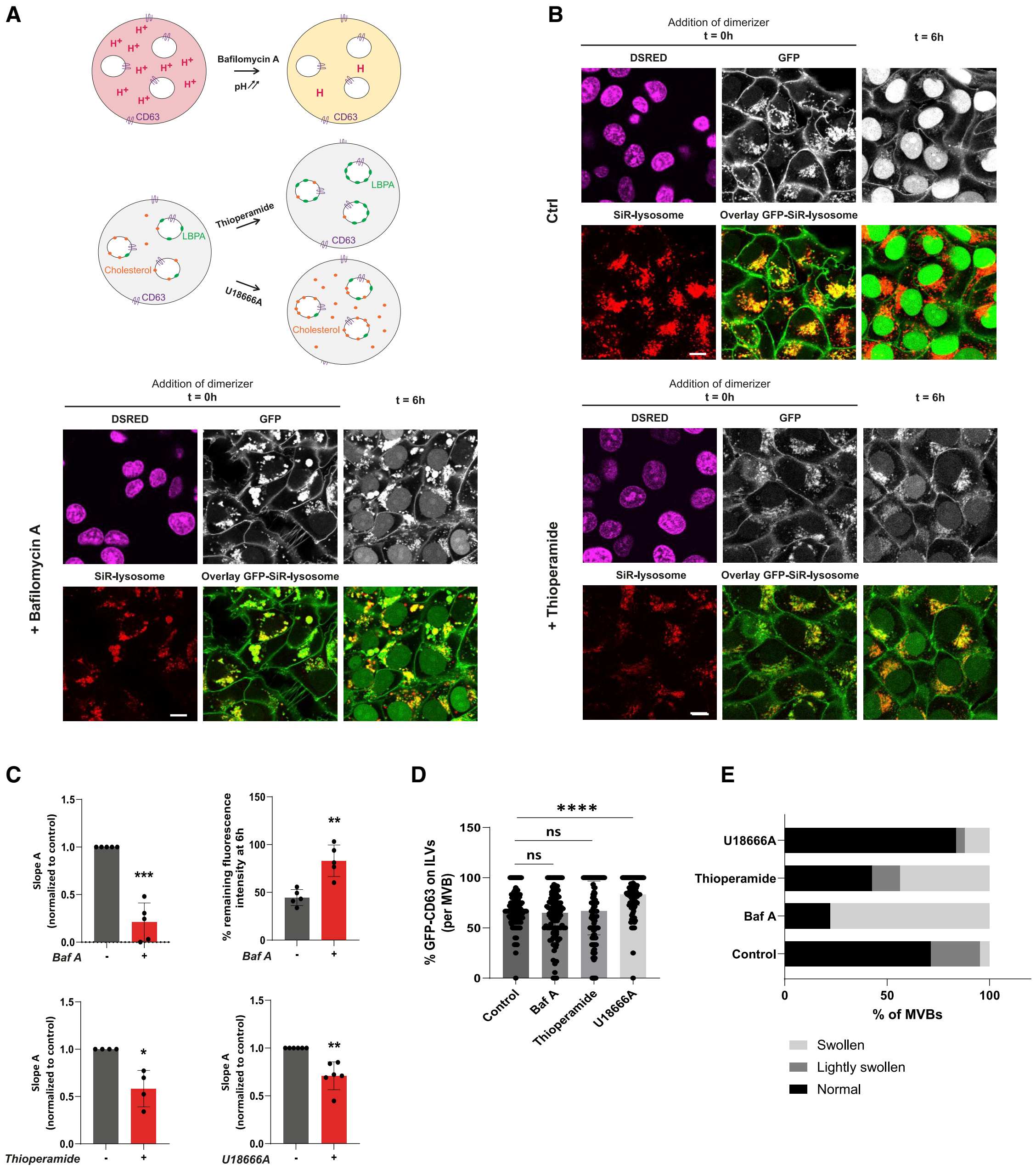

Figure 3. Alterations in MVB pH and lipid repertoire hamper ILV retrofusion

(A) Scheme summarizing the different treatments used to modulate membrane composition and dynamics within the MVB: (1) bafilomycin A (Baf A) blocks acidification; and (2) thioperamide and U18666A lead to accumulation of LBPA and cholesterol in MVBs, respectively.

(B) Effect(s) of Baf A and thioperamide treatments on the rate of ILV retrofusion. Representative confocal images of GFP-CD63 (white) distribution before (t = 0 h) and after (t = 6 h) treatment with dimerizer are shown, along with color overlays of GFP (green) with SiR-lysosome (red) in retrofusion-monitoring cells. DsRED-positive (magenta) nuclei indicate expression of the split TEV protease at t = 0. Scale bars, 10 μm.

(C) Quantification of slope A (paired t test) and the percentage of remaining fluorescence intensity at 6 h for the indicated conditions normalized to control. Shown is mean ± SD; n = 5 independent experiments for Baf A, n = 4 for thioperamide, and n = 6 for U18666A. See also Figure S2.

(D) Quantification of GFP-CD63 as detected by immunogold labeling on ILVs relative to the LM (expressed as ratio per MVB) following incubation with U18666A, thioperamide, or Baf A. Shown is median ± IQR from over 98 MVBs from n = 2 independent experiments.

(E) Quantification of MVB structure (normal, lightly swollen, or swollen) as expressed as a percentage of MVBs for each condition, U18666A, thioperamide, or Baf A. Shown are the relative results from n $\geq$ 98 MVBs from n = 2 independent experiments.

Statistical differences between the groups were assessed using paired or unpaired Student's t test (C), or Mann-Whitney test (D) (*p < 0.05, **p < 0.01, ***p < 0.001, ****p < 0.0001, ns = not significant). See also Figure S2.

CellPress
OPEN ACCESS

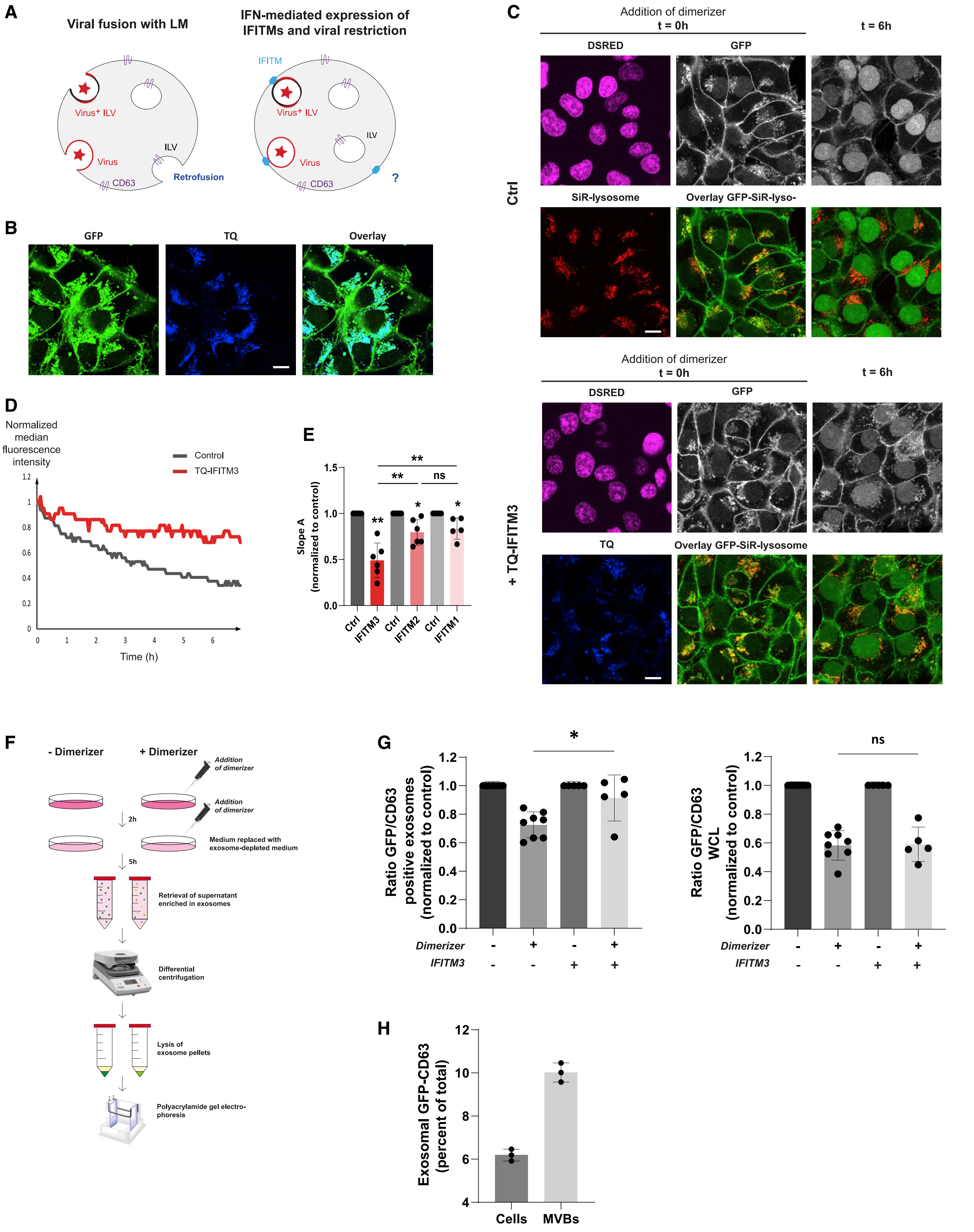

(legend on next page)

access the host's cytoplasm. Furthermore, type I interferons, induced upon viral infection, can stimulate expression of interferon-induced transmembrane proteins (IFITMs) that block viral escape from endosomes.[25] Specifically, IFITM3 expression has been shown to promote accumulation of cholesterol in MVBs of infected cells, hence impeding retrofusion of vesicular stomatitis virus (VSV)-positive ILVs.[26] We therefore considered whether stable introduction of TQ-IFITM1–3 (Figure 4A), all of which colocalized with GFP-CD63 in endolysosomal compartments (Figures 3 and 4), would affect the rate of constitutive retrofusion. GFP fluorescence decay was reduced by ~50% in the presence of TQ-IFITM3 (Figures 4C–4E) but was affected to a lesser extent by IFITM1 and IFITM2 (Figures 4E, S3A, and S3C). This difference is consistent with previous findings showing that IFITM3 expression restricts viral access to the host's cytoplasm more efficiently than expression of IFITM1 or 2.[27] It has been proposed that IFITM3 blocks viral membrane hemifusion[28] by affecting membrane curvature required during viral fusion.[29] Our data suggest that IFITM3 may hamper ILV retrofusion through a similar mechanism. Taken together with the effects of endosomal pH and ILV lipid content on retrofusion (Figure 3), the above observations reveal mechanistic parallels between ILV retrofusion and viral fusion with endosomal membranes and lend experimental support to the notion that some viruses exploit the former pathway to further their infection program, whereas certain antiviral host proteins can inhibit it at the cost of attenuated retrofusion.[30, 31]

Depending on their cargoes, ILVs can be expulsed into extracellular space following fusion of the LM with the plasma membrane.[2] These extracellular vesicles, or exosomes, can transfer ILV contents between cells; exosome transmission has been implicated in the pathogenesis of various diseases, including tumor metastasis and dissemination of prion-like proteins[5, 32, 33]; and retrofusion has been proposed as a key step in exosome uptake.[34] We took advantage of our cell-based system to explore a possible relationship between constitutive retrofusion and exosome release. The remaining fluorescence observed at the end of the GFP-CD63 decay following prolonged exposure to dimerizer (Figures 2B and 2C) implies that a proportion of ILVs remains inert to retrofusion. We therefore considered whether these ILVs constitute the reservoir from which exosomes arise. To test this, exosomes were isolated, using differential centrifugation, from the medium of cells cultured either in the presence or absence of dimerizer for 7 h (Figure 4F). Isolates were then examined by EM (Figure S4A), which revealed small vesicles 50–120 nm in diameter, corresponding to the expected size range for exosomes and ILVs.[35] To assess membrane purity, the exosome fraction was further analyzed by immunoblot, identifying the expected exosome markers CD63, major histocompatibility complex class II (MHC class II), and Tsg101 but not the LM resident LAMP2 or markers for the recycling endosome (transferrin receptor), endoplasmic reticulum (ER) (Calnexin), or trans-Golgi network (TGN) (Golgin97) (Figure S4B).

To determine the extent to which exosomes derive from the retrofusion-inert ILVs, the relative amount of GFP-CD63 present in exosome isolates derived from dimerizer-treated cells was compared to total (untagged) CD63 in the same samples (Figure S4C). In principle, exosomes originating from retrofusion-inert ILVs should maintain their GFP-CD63, whereas GFP would be removed from CD63 in the dynamic ILV pool susceptible to retrofusion. A 30% reduction in the ratio of GFP-positive exosomes over CD63-positive exosomes was observed as a function of treatment with dimerizer (Figure 4G), implying that at most a third of exosomes come from dynamic ILVs (which have lost their associated GFP), whereas the majority derive from inert ILVs (harboring intact GFP-CD63 not accessible to the cytosolic activated TEV protease during the harvesting period). To explore the existence of an equilibrium between these populations, we tested whether inhibiting retrofusion would promote exocytosis of affected ILVs. To this end, exosomes were isolated from the medium of control cells versus those ectopically expressing TQ-IFITM3 treated either in the presence or absence of dimerizer. In the case of the TQ-IFITM3-expressing cells, the abundance of GFP-CD63 in

Figure 4. Activation of antiviral response genes attenuates retrofusion of ILVs and renders a fraction of dynamic exosomes inert

(A) Schematic representation of the fusion of viruses with the LM of late endosomes and the potential function of IFITM proteins in restricting fusion and thus infection of host cells.

(B) Confocal images showing the localization of TQ-IFITM3 to late endocytic compartments. Fluorescence overlays of TQ-IFITM3 (blue) with GFP-CD63 (green) are shown, as indicated. Scale bar, 10 μm.

(C) Effect(s) of IFITM3 overexpression on the rate of ILV retrofusion. Representative confocal images of GFP-CD63 (white) distribution before (t = 0 h) and after (t = 6 h) treatment with dimerizer are shown, along with color overlays of GFP (green) with SiR-lysosome (red) in retrofusion-monitoring GFP-CD63 cells. DsRED-positive (magenta) nuclei indicate expression of the split TEV protease at t = 0. Scale bars, 10 μm.

(D) Representative plots of normalized late-endosome-associated median GFP fluorescence intensity over time (min) following dimerizer addition in control cells (gray line) versus those overexpressing TQ-IFITM3 (red line).

(E) Quantification of slope A for the indicated conditions normalized to control cells in the same experiment. Shown is mean ± SD from n = 6 independent experiments for TQ-IFITM3, n = 6 for TQ-IFITM2, and n = 5 for TQ-IFITM1. Statistical differences between each condition and its control were assessed using paired Student's t test and those between different conditions using unpaired Student's t test. See also Figure S3.

(F) Workflow for exosome isolation from retrofusion-monitoring cells.

(G) Quantification of the ratio of GFP over total CD63 signal in exosome fractions (left graph) and WCL (right graph) prepared following incubation of control cells or cells overexpressing TQ-IFITM3 and cultured in the presence (+) or absence (−) of dimerizer for 9 h. Shown is mean ± SD normalized to control from n > 5 independent experiments.

(H) Quantification of the percentage of GFP-CD63 secreted in exosomes relative to the total cell lysate where GFP-CD63 is expressed in MVBs and the plasma membrane. The relative contribution of GFP-CD63 fluorescence in MVBs was determined by 3D reconstitution of GFP fluorescence in cells (see Video S4). GFP-CD63 in exosomes was compared in a dilution series of GFP labeling as detected by WB analyses and the ratio of GFP fluorescence in the total cell versus MVB was determined by microscopy. Shown is the result of n = 3 independent exosome versus total lysate isolations assessed by WB. Shown is mean ± SD.

Statistical differences between groups were assessed using unpaired Student's t test unless otherwise indicated (*p < 0.05, **p < 0.01, ***p < 0.001, ****p < 0.0001, ns = not significant). See also Figures S3 and S4 and Video S4.

exosome isolates was normalized to both total (untagged) CD63 and TQ-IFITM3 in the same samples. Only a 10% decrease in the ratio of GFP-positive over CD63/IFITM3-positive exosomes was noted upon dimerizer treatment (Figure 4G and S4C), suggesting that IFITM3-mediated block of retrofusion renders at least a fraction of dynamic exosomes inert. Because our findings indicate that most exosomes are inert to retrofusion prior to exocytosis, it begs the questions of how retrofusion is controlled and what determines the fate of ILVs. Cytosolic factors, such as the endosomal sorting complexes required for transport (ESCRT)-associated protein Alix,[36] may contribute to intra-endosomal membrane dynamics to some extent, but cargo could also play an important role in dictating the ultimate ILV destination. Although retrofusion and exosome formation have some relationship, the exact molecular details still need to be determined in future studies.

Finally, we sought to exploit our chemically inducible cell-based system to assess the relative contribution of different fates experienced by ILVs at steady state (retrofusion, secretion, degradation). The numbers, however, represent a rough approximation as we follow the bulk of endosomes whereas particular endosomes may be more specialized in a specific pathway. Exosomes released over a 6-h period were isolated from cells and the amount of GFP-CD63 in exosomes was related to GFP-CD63 in total cell lysates (6%; Figures 4H and S4D–S4F), and further corrected for the distribution of GFP-CD63 in endosomes versus the plasma membrane (62% in MVB; Video S4). Around 10% of GFP-CD63 ended up in exosomes (Figure 4H). Along with pulse-chase experiment results (Figures 2G and 2H), we calculated the percentage of GFP-CD63 (from whole-cell lysate [WCL] or MVBs) that is either degraded in lysosomes or secreted in exosomes (Figure S4G). Considering these numbers, at least 11% of ILVs may participate in retrofusion in the 6 h of detection (Figure S4H). This number is likely higher because the TEV protease is not modifying every GFP-CD63 exposed to the cytosol. How individual ILVs differ to end up in one of the three different pathways is unclear, but is likely influenced by the type of substrate involved (e.g., epidermal growth factor receptor [EGFR] degradation upon EGF binding[37]).

In conclusion, the development and application of the first chemically tunable system to visualize and quantify the rate of ILV retrofusion in living cells have enabled us to show that constitutive retrofusion is a dynamic process occurring as part of the normal MVB lifestyle in unperturbed cells. However, only a part of the ILV population is able to fuse back with the limiting membrane of the MVB. In other words, MVBs harbor different pools of ILVs existing in equilibrium. One pool contributes to dynamics within MVBs and allows intraluminal proteins to return to the LM. The other, more inert, pool encompasses the bulk of secreted exosomes, which can transfer information to other cells, and the rest accounts for ILV cargo destined for lysosomal degradation. What distinguishes these different ILV pools is unclear at this point. Furthermore, we have observed a number of parallels between ILV retrofusion and viral entry, including the influence of pH and lipids, as well as the attenuating effects of the host antiviral protein IFITM3. Collectively, these findings support the notion that viruses employ the dynamic nature of MVBs to complete their infection cycle and expose a new twist on the emerging paradigm of pathogen-instigated subversion of host processes.

STAR★METHODS

Detailed methods are provided in the online version of this paper and include the following:

- KEY RESOURCES TABLE
- RESOURCE AVAILABILITY
  - Lead contact
  - Materials availability
  - Data and code availability
- EXPERIMENTAL MODEL AND SUBJECT DETAILS
  - Cell lines and culturing
- METHOD DETAILS
  - Antibodies and reagents
  - Constructs
  - Construction of the system
  - Confocal microscopy
  - Quantification of ILV retrofusion
  - DMM pulse-chase
  - Electron microscopy (EM)
  - Exosome isolation
  - Western blotting
- QUANTIFICATION AND STATISTICAL ANALYSIS

SUPPLEMENTAL INFORMATION

Supplemental information can be found online at https://doi.org/10.1016/j.cub.2021.06.022.

A video abstract is available at https://doi.org/10.1016/j.cub.2021.06.022#mmc7.

ACKNOWLEDGMENTS

We thank Dr. J. Wells (University of California) for providing the split TEV protease system, the LUMC microscopy facility for support, and members of the Neefjes group for critical discussions. This work was supported by an ERC Advanced Grant ERCOPE no. 694307 awarded to J.N. We thank Dr. K. Jalink (NKI) for valuable discussions and use of equipment.

AUTHOR CONTRIBUTIONS

J.N. and L.J. developed the concept; P.P. and J.N. designed the experiments; P.P. performed most of the experiments supported by constructs and cell lines generated by L.J.; H.J. and D.v.E. performed immunogold EM; L.M.V. and B.v.d.B. designed the macro for time-lapse analysis; J.N., P.P., and I.B. drafted the manuscript with input from all authors.

DECLARATION OF INTERESTS

The authors declare no competing interests.

Received: November 10, 2020
Revised: May 4, 2021
Accepted: June 9, 2021
Published: July 7, 2021

REFERENCES

1. Neefjes, J., Jongsma, M.M.L., and Berlin, I. (2017). Stop or go? Endosome positioning in the establishment of compartment architecture, dynamics, and function. Trends Cell Biol. *27*, 580–594.
2. Woodman, P.G., and Futter, C.E. (2008). Multivesicular bodies: co-ordinated progression to maturity. Curr. Opin. Cell Biol. *20*, 408–414.

3. Gruenberg, J. (2020). Life in the lumen: the multivesicular endosome. Traffic *21*, 76–93.

4. van der Goot, F.G., and Gruenberg, J. (2006). Intra-endosomal membrane traffic. Trends Cell Biol. *16*, 514–521.

5. Raposo, G., and Stoorvogel, W. (2013). Extracellular vesicles: exosomes, microvesicles, and friends. J. Cell Biol. *200*, 373–383.

6. Perrin, P., Jongsma, M.L., Neefjes, J., and Berlin, I. (2019). The labyrinth unfolds: architectural rearrangements of the endolysosomal system in antigen-presenting cells. Curr. Opin. Immunol. *58*, 1–8.

7. Kleijmeer, M., Ramm, G., Schuurhuis, D., Griffith, J., Rescigno, M., Ricciardi-Castagnoli, P., Rudensky, A.Y., Ossendorp, F., Melief, C.J., Stoorvogel, W., and Geuze, H.J. (2001). Reorganization of multivesicular bodies regulates MHC class II antigen presentation by dendritic cells. J. Cell Biol. *155*, 53–63.

8. Zwart, W., Griekspoor, A., Kuijl, C., Marsman, M., van Rheenen, J., Janssen, H., Calafat, J., van Ham, M., Janssen, L., van Lith, M., et al. (2005). Spatial separation of HLA-DM/HLA-DR interactions within MIIC and phagosome-induced immune escape. Immunity *22*, 221–233.

9. Wubbolts, R., Fernandez-Borja, M., Oomen, L., Verwoerd, D., Janssen, H., Calafat, J., Tulp, A., Dusseljee, S., and Neefjes, J. (1996). Direct vesicular transport of MHC class II molecules from lysosomal structures to the cell surface. J. Cell Biol. *135*, 611–622.

10. Wehr, M.C., Laage, R., Bolz, U., Fischer, T.M., Grünewald, S., Scheek, S., Bach, A., Nave, K.A., and Rossner, M.J. (2006). Monitoring regulated protein-protein interactions using split TEV. Nat. Methods *3*, 985–993.

11. Pols, M.S., and Klumperman, J. (2009). Trafficking and function of the tetraspanin CD63. Exp. Cell Res. *315*, 1584–1592.

12. Neefjes, J.J., Lindhout, J., Broxterman, H.J., van der Marel, G.A., van Boom, J.H., and Ploegh, H.L. (1989). Non-carrier-mediated uptake of the mannosidase I inhibitor 1-deoxymannojirimycin by K562 erythroleukemic cells. J. Biol. Chem. *264*, 10271–10275.

13. Engering, A., Kuhn, L., Fluitsma, D., Hoefsmit, E., and Pieters, J. (2003). Differential post-translational modification of CD63 molecules during maturation of human dendritic cells. Eur. J. Biochem. *270*, 2412–2420.

14. Escola, J.M., Kleijmeer, M.J., Stoorvogel, W., Griffith, J.M., Yoshie, O., and Geuze, H.J. (1998). Selective enrichment of tetraspan proteins on the internal vesicles of multivesicular endosomes and on exosomes secreted by human B-lymphocytes. J. Biol. Chem. *273*, 20121–20127.

15. Helenius, A. (2013). Virus entry: what has pH got to do with it? Nat. Cell Biol. *15*, 125.

16. Yoshimori, T., Yamamoto, A., Moriyama, Y., Futai, M., and Tashiro, Y. (1991). Bafilomycin A1, a specific inhibitor of vacuolar-type H(+)-ATPase, inhibits acidification and protein degradation in lysosomes of cultured cells. J. Biol. Chem. *266*, 17707–17712.

17. Bissig, C., and Gruenberg, J. (2013). Lipid sorting and multivesicular endosome biogenesis. Cold Spring Harb. Perspect. Biol. *5*, a016816.

18. Kobayashi, T., Stang, E., Fang, K.S., de Moerloose, P., Parton, R.G., and Gruenberg, J. (1998). A lipid associated with the antiphospholipid syndrome regulates endosome structure and function. Nature *392*, 193–197.

19. Gruenberg, J. (2003). Lipids in endocytic membrane transport and sorting. Curr. Opin. Cell Biol. *15*, 382–388.

20. Moreau, D., Vacca, F., Vossio, S., Scott, C., Colaco, A., Paz Montoya, J., Ferguson, C., Damme, M., Moniatte, M., Parton, R.G., et al. (2019). Drug-induced increase in lysobisphosphatidic acid reduces the cholesterol overload in Niemann-Pick type C cells and mice. EMBO Rep. *20*, e47055.

21. Lu, F., Liang, Q., Abi-Mosleh, L., Das, A., De Brabander, J.K., Goldstein, J.L., and Brown, M.S. (2015). Identification of NPC1 as the target of U18666A, an inhibitor of lysosomal cholesterol export and Ebola infection. eLife *4*, e12177.

22. Stoeck, I.K., Lee, J.Y., Tabata, K., Romero-Brey, I., Paul, D., Schult, P., Lohmann, V., Kaderali, L., and Bartenschlager, R. (2017). Hepatitis C virus replication depends on endosomal cholesterol homeostasis. J. Virol. *92*, e01196-17.

23. Kühnl, A., Musiol, A., Heitzig, N., Johnson, D.E., Ehrhardt, C., Grewal, T., Gerke, V., Ludwig, S., and Rescher, U. (2018). Late endosomal/lysosomal cholesterol accumulation is a host cell-protective mechanism inhibiting endosomal escape of influenza A virus. mBio *9*, e01345-18.

24. Le Blanc, I., Luyet, P.P., Pons, V., Ferguson, C., Emans, N., Petiot, A., Mayran, N., Demaurex, N., Fauré, J., Sadoul, R., et al. (2005). Endosome-to-cytosol transport of viral nucleocapsids. Nat. Cell Biol. *7*, 653–664.

25. Shi, G., Schwartz, O., and Compton, A.A. (2017). More than meets the I: the diverse antiviral and cellular functions of interferon-induced transmembrane proteins. Retrovirology *14*, 53.

26. Amini-Bavil-Olyaee, S., Choi, Y.J., Lee, J.H., Shi, M., Huang, I.C., Farzan, M., and Jung, J.U. (2013). The antiviral effector IFITM3 disrupts intracellular cholesterol homeostasis to block viral entry. Cell Host Microbe *13*, 452–464.

27. Bailey, C.C., Zhong, G., Huang, I.C., and Farzan, M. (2014). IFITM-family proteins: the cell's first line of antiviral defense. Annu. Rev. Virol. *1*, 261–283.

28. Li, K., Markosyan, R.M., Zheng, Y.M., Golfetto, O., Bungart, B., Li, M., Ding, S., He, Y., Liang, C., Lee, J.C., et al. (2013). IFITM proteins restrict viral membrane hemifusion. PLoS Pathog. *9*, e1003124.

29. Ling, S., Zhang, C., Wang, W., Cai, X., Yu, L., Wu, F., Zhang, L., and Tian, C. (2016). Combined approaches of EPR and NMR illustrate only one transmembrane helix in the human IFITM3. Sci. Rep. *6*, 24029.

30. Desai, T.M., Marin, M., Chin, C.R., Savidis, G., Brass, A.L., and Melikyan, G.B. (2014). IFITM3 restricts influenza A virus entry by blocking the formation of fusion pores following virus-endosome hemifusion. PLoS Pathog. *10*, e1004048.

31. Gruenberg, J., and van der Goot, F.G. (2006). Mechanisms of pathogen entry through the endosomal compartments. Nat. Rev. Mol. Cell Biol. *7*, 495–504.

32. Tkach, M., and Théry, C. (2016). Communication by extracellular vesicles: where we are and where we need to go. Cell *164*, 1226–1232.

33. Howitt, J., and Hill, A.F. (2016). Exosomes in the pathology of neurodegenerative diseases. J. Biol. Chem. *291*, 26589–26597.

34. Joshi, B.S., de Beer, M.A., Giepmans, B.N.G., and Zuhorn, I.S. (2020). Endocytosis of extracellular vesicles and release of their cargo from endosomes. ACS Nano *14*, 4444–4455.

35. Gurunathan, S., Kang, M.H., Jeyaraj, M., Qasim, M., and Kim, J.H. (2019). Review of the isolation, characterization, biological function, and multifarious therapeutic approaches of exosomes. Cells *8*, 307.

36. Bissig, C., and Gruenberg, J. (2014). ALIX and the multivesicular endosome: ALIX in Wonderland. Trends Cell Biol. *24*, 19–25.

37. Bakker, J., Spits, M., Neefjes, J., and Berlin, I. (2017). The EGFR odyssey—from activation to destruction in space and time. J. Cell Sci. *130*, 4087–4096.

38. Johnson, J.P., Demmer-Dieckmann, M., Meo, T., Hadam, M.R., and Riethmüller, G. (1981). Surface antigens of human melanoma cells defined by monoclonal antibodies. I. Biochemical characterization of two antigens found on cell lines and fresh tumors of diverse tissue origin. Eur. J. Immunol. *11*, 825–831.

39. Blott, E.J., Bossi, G., Clark, R., Zvelebil, M., and Griffiths, G.M. (2001). Fas ligand is targeted to secretory lysosomes via a proline-rich domain in its cytoplasmic tail. J. Cell Sci. *114*, 2405–2416.

40. Gray, D.C., Mahrus, S., and Wells, J.A. (2010). Activation of specific apoptotic caspases with an engineered small-molecule-activated protease. Cell *142*, 637–646.

41. Vennegoor, C., and Rümke, P. (1986). Circulating melanoma-associated antigen detected by monoclonal antibody NKI/C-3. Cancer Immunol. Immunother. *23*, 93–100.

42. Rocha, N., Kuijl, C., van der Kant, R., Janssen, L., Houben, D., Janssen, H., Zwart, W., and Neefjes, J. (2009). Cholesterol sensor ORP1L contacts the

CellPress
OPEN ACCESS

ER protein VAP to control Rab7-RILP-p150 Glued and late endosome positioning. J. Cell Biol. *185*, 1209–1225.

43. Schindelin, J., Arganda-Carreras, I., Frise, E., Kaynig, V., Longair, M., Pietzsch, T., Preibisch, S., Rueden, C., Saalfeld, S., Schmid, B., et al. (2012). Fiji: an open-source platform for biological-image analysis. Nat. Methods *9*, 676–682.

44. Chen, G., and Deng, X. (2018). Cell synchronization by double thymidine block. Bio. Protoc. *8*, e2994.

45. Calafat, J., Janssen, H., Ståhle-Bäckdahl, M., Zuurbier, A.E., Knol, E.F., and Egesten, A. (1997). Human monocytes and neutrophils store transforming growth factor-alpha in a subpopulation of cytoplasmic granules. Blood *90*, 1255–1266.

STAR★METHODS

KEY RESOURCES TABLE

REAGENT or RESOURCE	SOURCE	IDENTIFIER
Antibodies		
Mouse anti-CD63 NKI-C3	NKI	N/A
rabbit anti-GFP	NKI	N/A
HRP-goat anti-Mouse IgG (H+L)	ThermoFisher Scientific	Cat#G21040; RRID:AB_2536527
HRP-goat anti-rabbit IgG (H+L)	ThermoFisher Scientific	Cat# G21234; RRID:AB_2536530
Mouse monoclonal Lamp2 antibody	SantaCruz	Cat# sc-18822; RRID:AB_626858
Rabbit monoclonal calnexin antibody	Cell Signaling Technology	Cat#2679; RRID:AB_2228381
Mouse monoclonal transferrin receptor antibody	ThermoFisher Scientific	Cat#13-6800; RRID:AB_2533029
Mouse monoclonal HLA-DR antibody	NKI	N/A
Mouse monoclonal Tsg101 antibody	ThermoFisher Scientific	MA1-23296; RRID:AB_561859
Chemicals, peptides, and recombinant proteins		
1-Deoxymannojirimycin hydrochloride	Sigma-Aldrich	73465-43-7
Thioperamide maleate	Cayman Chemical	3039-71-2
U18666A	Cayman Chemical	148440-81-7
Bafilomycin A1	Tebu-Bio	88899-55-2
Heterodimerizer	Takara	
SiR-Lysosome	Tebu-Bio	SC012
Experimental models: Cell lines		
Melanoma cell line MelJuso	Johnson et al.[38]	database of the DSMZ MEL-JUSO
Oligonucleotides		
NLS mGFP fwd NheI CCCAGCT AGCGCCACCATGGTGAAACGACC AGCAGCAACAAAGAAAGCAGGAC AAGCAAAGAAAAAGAAGATGGTG AGCAAGGGCGAGGAG	This paper	N/A
GFP ENLYFQS rev bglII CCCAAGAT CTACTCTGGAAATACAGATTTTCCC CGCCCCCCTTGTACAGCTCGTCCAT	This paper	N/A
C-TEV 119-242 fwd NheI CCCAGCTAGCAAGAGCATGTCTAGCATGGT	This paper	N/A
C-TEV 119-242 rev BamHI CCCAGGATCCTCATTGCGAGTACACCAATT	This paper	N/A
N-TEV 1-118 fwd SpeI CCCAACTAGTGGAGAAAGCTTGTTTAAGGG	This paper	N/A
N-TEV 1-118 rev BamHI CCCAGGATCCTTAAGCTTGGAAGTTGGTTG	This paper	N/A
F2A Fwd 1 GATCCAAGCGCGGAAAGCCAATTCCAAAC CCTCTTTTGGGCC	This paper	N/A
F2A Fwd 2 TCGACAGTACATCGGGATCAGGA GCGCCCGTGAAACAGACATTGAACTTCGAC CTTTTGAAGCTAGCAGGGGATGTCGAGTC GAACCCTGGACCAG	This paper	N/A
F2A Rev 1 GATCCTGGTCCAGGGTTCGACT CGACATCCCCTGCTAGCTTCAAAAGGTCG AAGTTCAATGTCTG	This paper	N/A
F2A Rev 2 TTTCACGGGCGCTCCTGATCCCG ATGTACTGTCGAGGCCCAAAAGAGGGTTTG GAATTGGCTTTCCGCGCTTG	This paper	N/A
IFITM1 fwd HinDIII CCCAAGCTTCGATGCACAAGGAG	This paper	N/A

(Continued on next page)

Continued

REAGENT or RESOURCE	SOURCE	IDENTIFIER
IFITM1 rev BamHI CCCAGGATCCCTAGTAACCCCGTT	This paper	N/A
IFITM2 fwd HinDIII CCCAAAGCTTCGATGAACCACATTGTGCAAAC	This paper	N/A
IFITM2 rev BamHI CCCAGGATCCCTATCGCTGGGCCTGGAC	This paper	N/A
IFITM3_HindIII_fwd CCCAAAGCTTCGATGAATCACACTGTCCAAACC	This paper	N/A
IFITM3_BamHI_rev CCCAGGATCCCTATCCATAGGCCTGGAAGATC	This paper	N/A
Recombinant DNA		
pCD63-EGFP-bos	Blott et al.[39]	N/A
FKBP N-TEV and FRB C-TEV	Gray et al.[40]	N/A
Software and algorithms		
LAS X	Leica Microsystems	https://www.leica-microsystems.com/
Fiji 1.52p	National Institutes of Health	https://imagej.net/Fiji/Downloads
Microsoft Excel	Microsoft Inc.	N/A
Adobe Illustrator CC 2018	Adobe Inc.	N/A
GraphPad Prism 8.4.2	GraphPad	https://www.graphpad.com/

RESOURCE AVAILABILITY

Lead contact

Further information and requests for resources and reagents should be directed to and will be fulfilled by the lead contact, Jacques Neefjes (j.j.c.neefjes@lumc.nl).

Materials availability

All unique/stable reagents generated in this study are available from the Lead Contact without restriction.

Data and code availability

- All data reported in this paper will be shared by the lead contact upon request.
- This paper does not report original code.
- Any additional information required to reanalyze the data reported in this paper is available from the lead contact upon request.

EXPERIMENTAL MODEL AND SUBJECT DETAILS

Cell lines and culturing

Cell Line Authentication was performed by Eurofins Genomics. MelJuso (human melanoma)[38] cells were cultured in IMDM (GIBCO) supplemented with 7.5% fetal calf serum (FCS, Greiner) at 37°C. For exosome isolation, cells were cultured in IMDM (GIBCO) supplemented with 9% exosome-depleted fetal bovine serum (ThermoFisher Scientific). The cell-based system was constructed in the following way. First, NLS-GFP-TCS-CD63 was transfected into MelJuSo cells (Effectene, QIAGEN). Following selection on G418 for stable expression, cells were retrovirally transduced with the split sniper TEV viral supernatant (protocol from Retroviral systems; Nolan lab) and selected on Puromycin. After single-cell sorting of double positive cells (GFP/DsRed), cells were screened for activation of the TEV protease in response to dimerizer. Clones demonstrating cleavage of GFP upon dimerizer addition were selected for the study. Where appropriate, Turq-IFITM1-3 was introduced by transfection (Effectene, QIAGEN) and, following selection on Hygromycin, triple positive (Turq/GFP/DsRED) cells were sorted and expanded for further experiments.

METHOD DETAILS

Antibodies and reagents

Mouse anti-CD63 NKI-C3[41] and rabbit anti-GFP,[42] followed respectively by HRP-goat anti-Mouse and HRP-goat anti-rabbit IgG (H+L) secondary antibodies (ThermoFisher Scientific) were used for detection of endogenous or overexpressed proteins by SDS-PAGE and western blot. Mouse monoclonal Lamp2 antibody was purchased from SantaCruz, rabbit monoclonal calnexin antibody was purchased from Cell Signaling Technology, mouse monoclonal transferrin receptor antibody and mouse monoclonal Tsg101

antibody were purchased from ThermoFisher Scientific, mouse monoclonal HLA-DR antibody (1B5) was obtained from the NKI, Amsterdam NL, SiR-Lysosome was purchased from Tebu-Bio (used at 50 μM for live cell imaging). Heterodimerizer (i.e., dimerizer) was purchased from Takara (0.35μM for live cell imaging, 0.5μM for exosome isolation). 1-Deoxymannojirimycin hydrochloride (DMM) and thymidine were purchased from Sigma-Aldrich. Thioperamide maleate and U18666A were purchased from Cayman Chemical (10 μM for live cell imaging, and 3 μg/mL respectively). Bafilomycin A1 was purchased from Tebu-Bio (100 nM for live cell imaging).

Constructs

The NLS-GFP-TCS-CD63 construct was generated by PCR adding a NLS (MVKRPAATKKAGQAKKKK) at the 5′ end of GFP and a TEV cleavage (ENLYFQS) moiety at 3′ end and this replaced the GFP part in the original GFP-CD63 construct.[39] The split sniper TEV was build up by dividing the FKBP N-TEV and FRB C-TEV[40] by a F2A motif allowing both parts to be expressed separately and at relative equal amounts under the same promotor. This box was cloned into a pMX Puro IRES2 NLS DsRed2 plasmid. The templates for IFITM1-3 were isolated from the Gateway pDONR223 (Entry ORF Library) library. cDNA's were first cloned into the mTurq2-C1 vector before being subcloned into pcDNA3.1 Hygro (Invitrogen). All constructs were sequence verified.

Construction of the system

The cell-based system was constructed in the following way. First, NLS-GFP-TCS-CD63 was transfected into MelJuSo cells (Effectene, QIAGEN). Following selection on G418 for stable expression, cells were retrovirally transduced with the split sniper TEV viral supernatant (protocol from Retroviral systems Nolan lab) and selected on Puromycin. After single-cell sorting of double positive cells (GFP/DsRed), cells were screened on activation of the TEV protease in response to dimerizer. Clones demonstrating cleavage of GFP upon dimerizer addition were used in the study. Where appropriate, Turq-IFITM1-3 was introduced by transfection (Effectene, QIAGEN) and, following selection on Hygromycin, triple positive (Turq/GFP/DsRED) cells were sorted and expanded for further experiments.

Confocal microscopy

Live cells were incubated with SiR-Lysosome (50 μM) for at least 30 min. The medium was removed and medium at a pH of 6.3 (adjusted with acetic acid 10mM in water) was added to the cells. Samples were imaged using a Leica SP8 WLL confocal microscope, HC PL APO 63x/1.40 oil immersion objective and HyD detectors. For Z stack imaging, a Leica SP8 with Andor Dragonfly spinning disc module was used, and 0,2 μM Z stacks were acquired with a 63x oil immersion objective. The microscope was equipped with a humidified climate control system at 37°C supplemented with 5% CO2. Images were collected using a digital zoom of 1.0 in 512 by 512 scanning format with line averaging [4x], at a rate of 180 s per frame for a period of at least 6 h. In the case of thioperamide or U18666A treatment, the cells were incubated with the compounds for 19h and 24h respectively before imaging. Cells were treated with Bafilomycin A1 for 6h before imaging.

Quantification of ILV retrofusion

Post-collection image processing and analysis were performed using Fiji.[43] A macro was programmed to analyze fluorescence timelapses (the different steps are shown in Video S2). Briefly, the macro was designed such that the user first sets an intensity threshold to define the area of endolysosomes (EL) based on SiR-lysosome fluorescence in the first and last frame, and the threshold for the other frames is automatically interpolated to allow for a possible slight fluctuation in fluorescence intensity. This allows quantification of the GFP signal in EL. The segmentation of nuclei (N) is achieved by thresholding the DSRED fluorescence, this allows quantification of the GFP signal in nuclei. Next, to define the area of the plasma membrane, a mask is created that segments the area outside of the nuclei and EL. Within this mask, the user can set a threshold on the GFP signal in the first and last frame to define the area of the plasma membrane. This allows quantification of the GFP signal in the plasma membrane (PM). The GFP fluorescence in the cytosol is then calculated by masking out all previous segmentations (EL, N, PM). Finally, the numeric results are stored in a table and the mean and median GFP fluorescence intensities in EL, N, PM and cytosol, and in the cytosol alone (background) are plotted overtime in a single graph. The median fluorescence intensities are then further analyzed in Excel. The median GFP background in the cytosol is subtracted from the median GFP signal in EL, and the resulting median GFP fluorescence in EL is normalized to its initial value (first frame).

DMM pulse-chase

Cells were synchronized by a double thymidine block,[44] and incubated with 1mM 1-deoxymannojirimycin (DMM) for the times indicated and then lysed in lysis buffer containing 0.5% NP-40, 150 mM NaCl, 50 mM Tris-HCl pH 7.6 and 5 mM $MgCl_2$ for 30 min before addition of Laemmli Sample Buffer (containing 80 mM DTT) followed by 10 min incubation at 95°C.

Electron microscopy (EM)

MelJuso cells were fixed for 2 hours in 2% paraformaldehyde + 0,2% glutaraldehyde in 0.1 M PHEM buffer (60 mM PIPES, 25 mM HEPES, 2 mM $MgCl_2$, 10 mM EGTA, pH 6.9) and then processed for ultrathin cryosectioning, as previously described.[45] Briefly, 50 nm cryosections were cut at −120°C using diamond knives in a cryoultramicrotome (Leica Aktiengesellschaft) and transferred with a mixture of sucrose and methylcellulose onto formvar-coated copper grids. The grids were placed on 35-mm Petri dishes containing 2% gelatine. Ultrathin frozen sections were incubated at room temperature with primary antibody and then incubated with 10-nm

CellPress
OPEN ACCESS

protein A-conjugated colloidal gold (Klumperman Lab, Utrecht University), as described.[45] After washing, the sections were fixed for 10 minutes in 1% glutaraldehyde, blocked and incubated with the second primary antibody and the second label protein A/15 nm gold. The sections were embedded in a mixture of methylcellulose and uranyl acetate and examined with a Philips CM10 electron microscope (FEI).

In case of exosome imaging, exosome-containing pellets were adsorbed onto formvar and carbon-coated copper grids after which they were negatively stained and embedded in a mixture of methylcellulose and uranyl acetate. Exosomes were imaged with a Tecnai 12 transmission electron microscope (FEI) at 120 kV acceleration voltage.

Exosome isolation

Cells were cultured in presence or absence of dimerizer for 2h, then incubated in exosome-depleted medium in the presence (or absence) of dimerizer for 5h. The supernatant (enriched in exosomes) was retrieved and subjected to differential centrifugation at 4°C: 1000 g for 10 min, then 2000 g for 20 min, 10000 g for 30 min and 100300 g for 2h. The exosome pellets were lysed in lysis buffer containing 0.5% NP-40, 150 mM NaCl, 50 mM Tris-HCl pH 7.6 and 5 mM $MgCl_2$ for 30 min before addition of Laemmli Sample Buffer (containing 80 mM DTT) followed by 10 min incubation at 95°C. After collection of the medium for exosome isolation, the cells were lysed as described above.

Western blotting

Samples were separated using 8% acrylamide gels and transferred to a PVDF membrane (Immobilon-P, 0.45μm, Millipore) at 100V for 3h. The membranes were blocked in PBS/5% Skim Milk (Oxiod) and incubated with a primary antibody for 1h diluted in PBS/0.1% Tween20 (Sigma-Aldrich) /5% Milk, washed three times for 10min in PBS/0.1% Tween and incubated with the secondary antibody for 1h diluted in PBS/0.1%Tween/5% Milk and washed three times again in PBS/0.1% Tween. Signals were detected on the Chemidoc XRS+ imager (Bio-Rad) using ECL (SuperSignal West Dura Extended Duration Substrate, Thermo Scientific). Intensity of bands was quantified using ImageJ.

QUANTIFICATION AND STATISTICAL ANALYSIS

Statistical analyses were performed with GraphPad Prism 7. All tests are mentioned in the corresponding figure. All experiments were performed independently at least 3 times, as indicated. All graphical data plots were produced using GraphPad Prism 7 and fons were adjusted with Adobe Illustrator CC.

Current Biology, Volume *31*

Supplemental Information

Retrofusion of intralumenal MVB membranes parallels viral infection and coexists with exosome release

Priscillia Perrin, Lennert Janssen, Hans Janssen, Bram van den Broek, Lennard M. Voortman, Daphne van Elsland, Ilana Berlin, and Jacques Neefjes

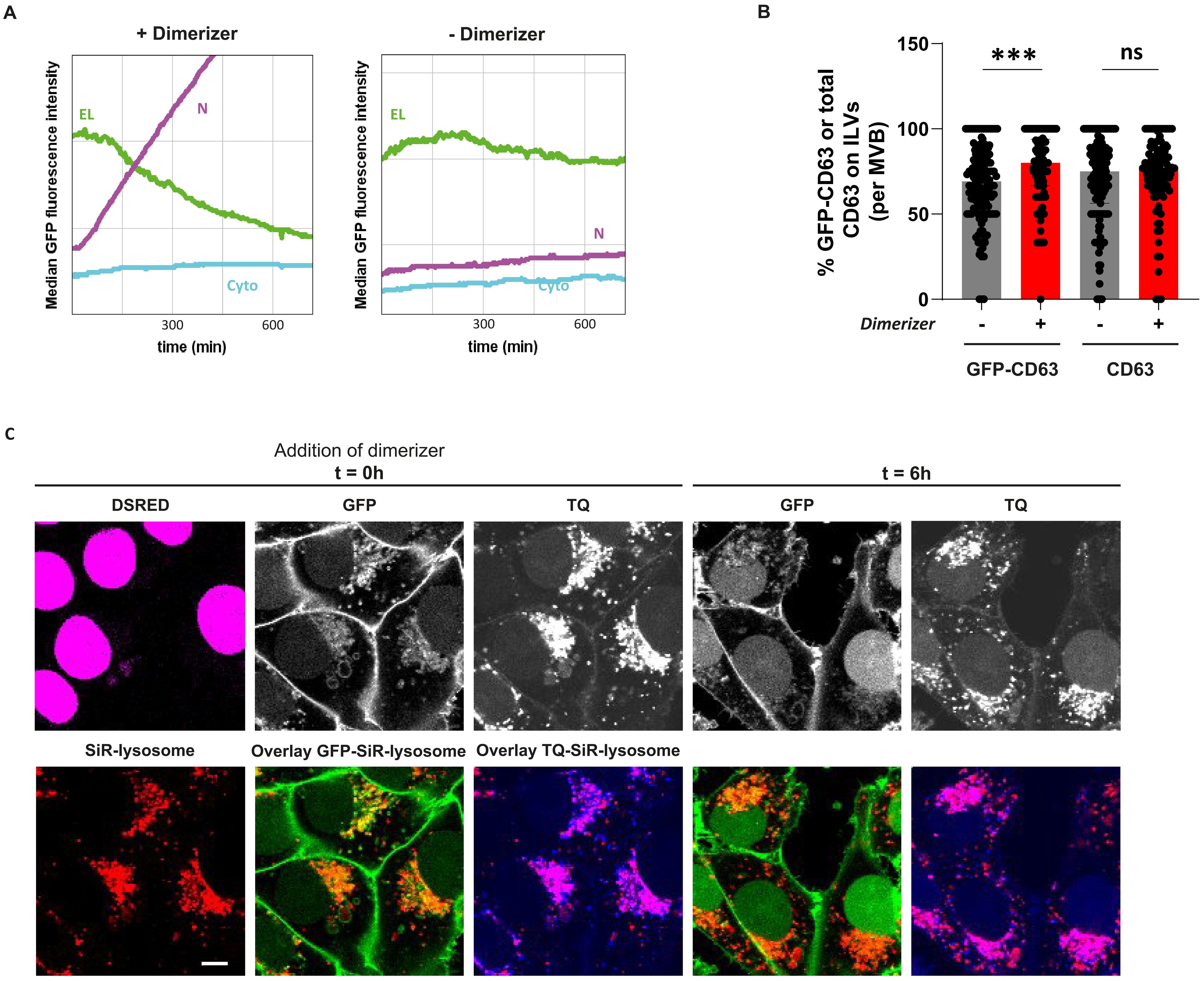
A
+ Dimerizer
- Dimerizer
Median GFP fluorescence intensity
EL
N
Cyto
300
600
time (min)
B
% GFP-CD63 or total CD63 on ILVs (per MVB)
150
100
50
0
***
ns
Dimerizer
-
+
GFP-CD63
CD63
C
Addition of dimerizer
t = 0h
t = 6h
DSRED
GFP
TQ
SiR-lysosome
Overlay GFP-SiR-lysosome
Overlay TQ-SiR-lysosome

Figure S1. Analysis of retrofusion measurement and TEV protease efficiency. Related to Figures 1 and 2.

(A) Representative plot of intracellular median GFP fluorescence intensity from GFP-CD63 cells either (+) or not (-) cultured with dimerizer for the time indicated (min) N: nucleus; EL late endosomes as labelled by SiR-lysosome; Cyto: cytoplasm. EL: endolysosome; N: nucleus; PM: plasma membrane; Cyto: cytoplasm.

(B) Quantification of GFP-CD63 as detected by immunogold labeling on ILVs relative to LM (expressed as ratio per MVB) following incubation with dimerizer (6h). Shown is median +/- IQR from over 98 MVBs from 2 independent experiments.

(C) Analysis of Lamp1-TEV-TQ expression in GFP-CD63 retrofusion-monitoring cells stained with SiR-lysosome. Confocal fluorescence images of GFP-CD63 and Lamp1-TEV-TQ (*white*) distribution before (t=0h) and after (t=5h) treatment with dimerizer are shown, along with color overlays of GFP (*green*) or TQ (blue) with SiR-Lysosome (*red*). NLS-DsRED (*magenta*) in the nucleus indicate expression of the split TEV protease at t=0. Bar: 10 µm. TQ : mTurquoise2.

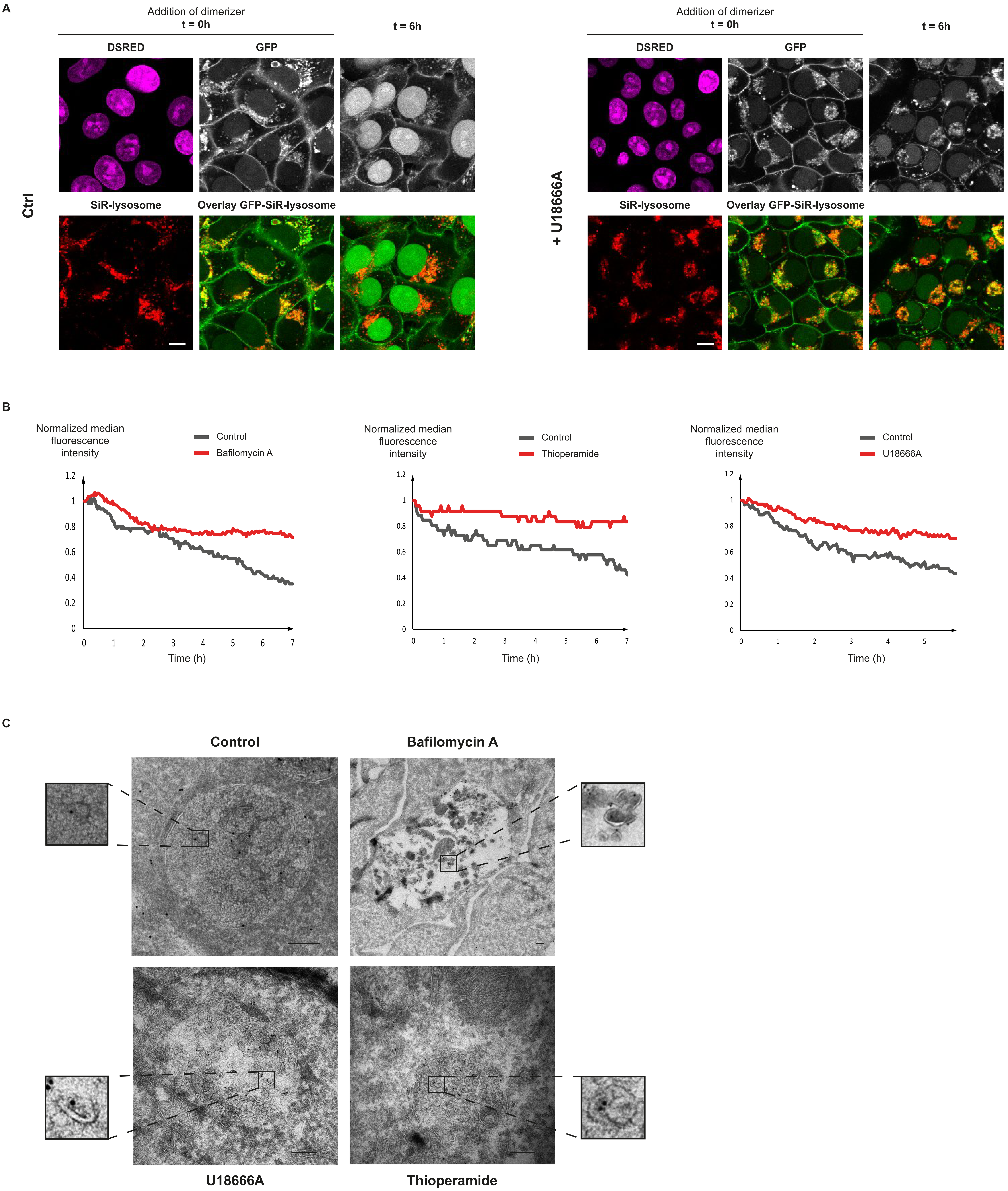
A
Addition of dimerizer
t = 0h
t = 6h
DSRED
GFP
SiR-lysosome
Overlay GFP-SiR-lysosome
Ctrl
+ U18666A
B
Normalized median fluorescence intensity
Control
Bafilomycin A
Thioperamide
U18666A
Time (h)
C
Control
Bafilomycin A
U18666A
Thioperamide

Figure S2. Effects of lipid and pH manipulation on the rate of ILV retrofusion. Related to Figure 3.

(A) Confocal images of control (left panel; Ctrl) and U18666A treated (right panel) GFP-CD63 cells as used for monitoring retrofusion. Confocal fluorescence images of GFP-CD63 (*white*) distribution before (t=0h) and after (t=6h) treatment with dimerizer are shown, along with color overlays of GFP (*green*) with SiR-Lysosome (*red*). NLS-DsRED (*magenta*) in the nucleus indicate expression of the split TEV protease at t=0. All scale bars, 10 µm.

(B) Representative plots of normalized median GFP fluorescence intensity in endolysosomes (as stained with SiR-lysosome) over time (min) following dimerizer addition in control cells (*gray*) versus those treated with Bafilomycin A, thioperamide or U18666A (*red*).

(C) Electron micrograph featuring immunogold labeling with GFP antibody of control cells following incubation with U18666A, thioperamide or Bafilomycin A. Scale bar, 200 nm.

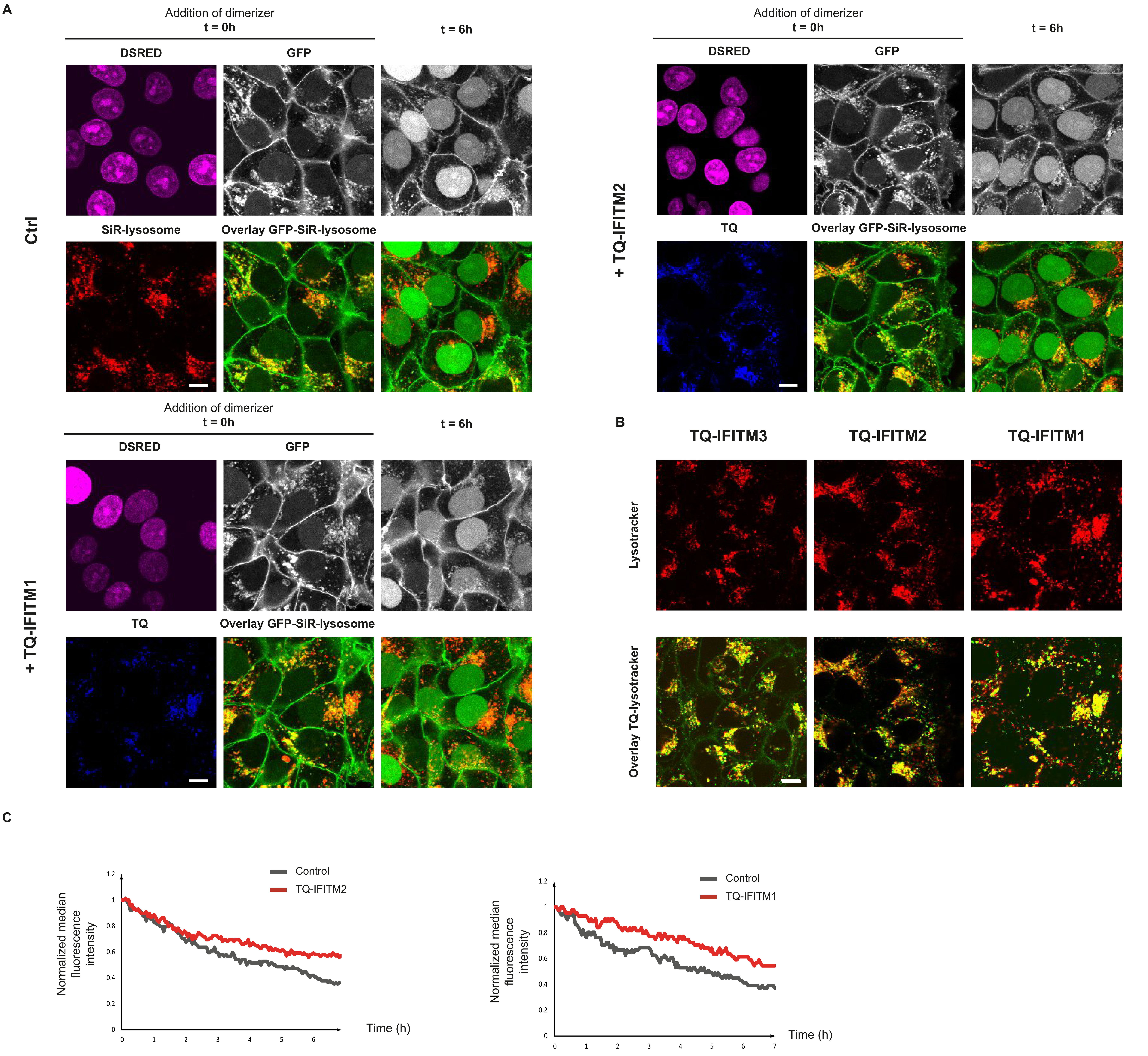
A
Addition of dimerizer
t = 0h
t = 6h
DSRED
GFP
Ctrl
SiR-lysosome
Overlay GFP-SiR-lysosome
Addition of dimerizer
t = 0h
t = 6h
DSRED
GFP
+ TQ-IFITM2
TQ
Overlay GFP-SiR-lysosome
Addition of dimerizer
t = 0h
t = 6h
DSRED
GFP
+ TQ-IFITM1
TQ
Overlay GFP-SiR-lysosome
B
TQ-IFITM3
TQ-IFITM2
TQ-IFITM1
Lysotracker
Overlay TQ-lysotracker
C
Normalized median fluorescence intensity
Control
TQ-IFITM2
Time (h)
Normalized median fluorescence intensity
Control
TQ-IFITM1
Time (h)

Figure S3. Effects of overexpression of the IFITM family members on the rate of ILV retrofusion. Related to Figure 4.

(A) Confocal images showing overexpression of IFITM1 or 2 in GFP-CD63 retrofusion-monitoring cells, as used for the retrofusion experiments. Confocal fluorescence images of GFP-CD63 (*white*) distribution before (t=0h) and after (t=6h) treatment with dimerizer are shown, along with color overlays of GFP (*green*) with SiR-Lysosome (*red*). NLS-DsRED in the nucleus (*magenta*) and TQ-IFITM1 and 2 (*blue*) respectively indicate expression of the split TEV protease and the IFITM proteins at t=0. Scale bars, 10 µm

(B) Localization of TQ-IFITM1, 2 and 3 proteins to late endocytic compartments as marked by SiR-Lysosome. Confocal fluorescence overlays of TQ-IFITM1-3 (*green*) with SiR-Lysosome (*red*) are shown in the bottom panel.

(C) Representative plots of normalized median GFP fluorescence intensity in endolysosomes (as marked by SiR-Lysosomes) over time (min) following dimerizer addition in control cells (*gray*) versus those overexpressing TQ-IFITM1 or 2 (*red*).

A

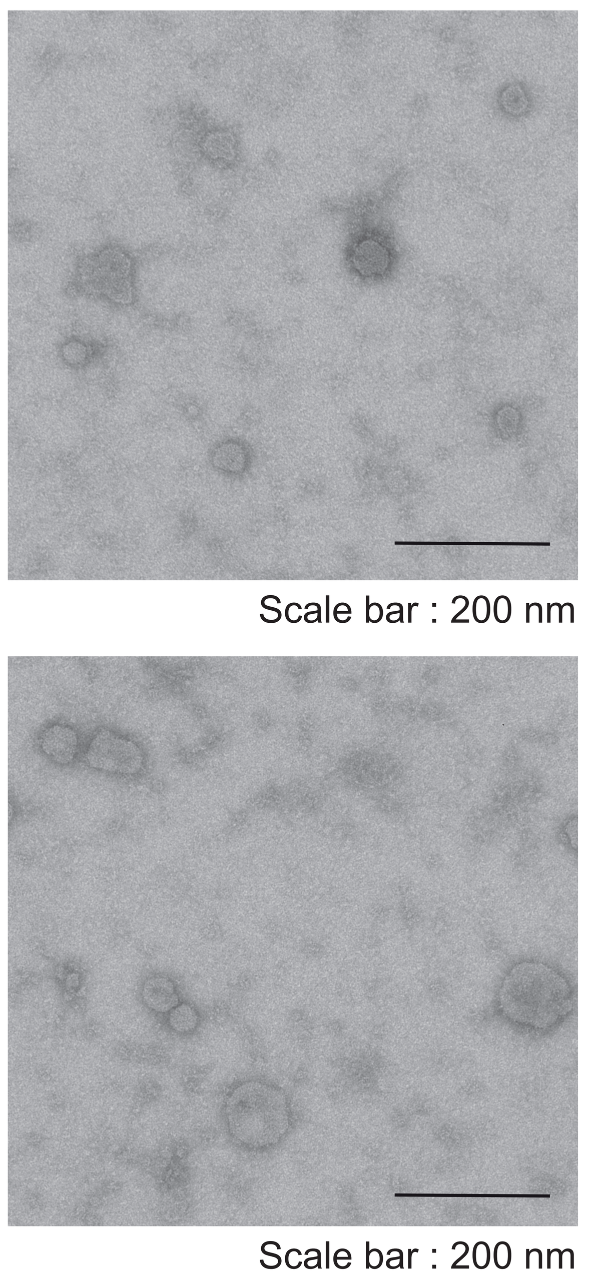

B

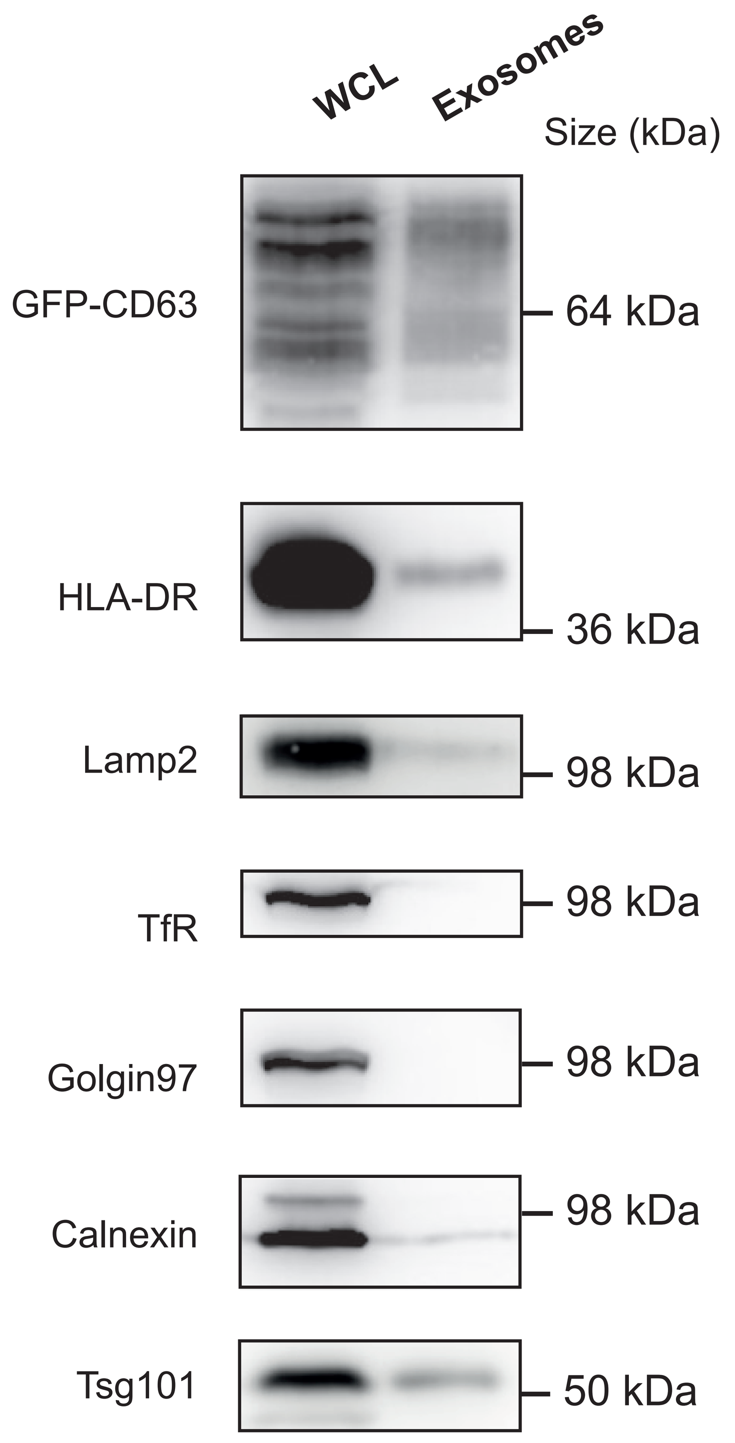

C

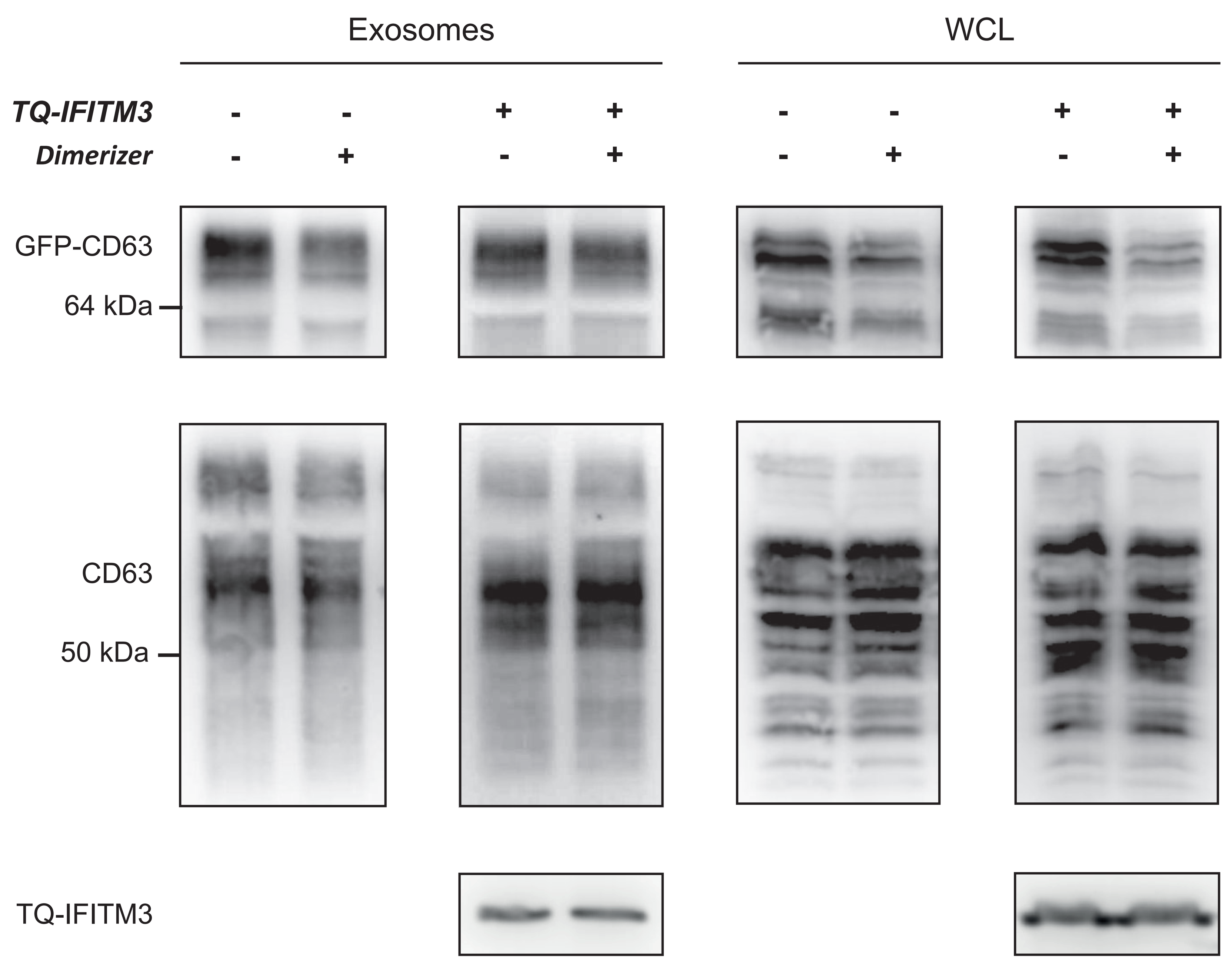

D

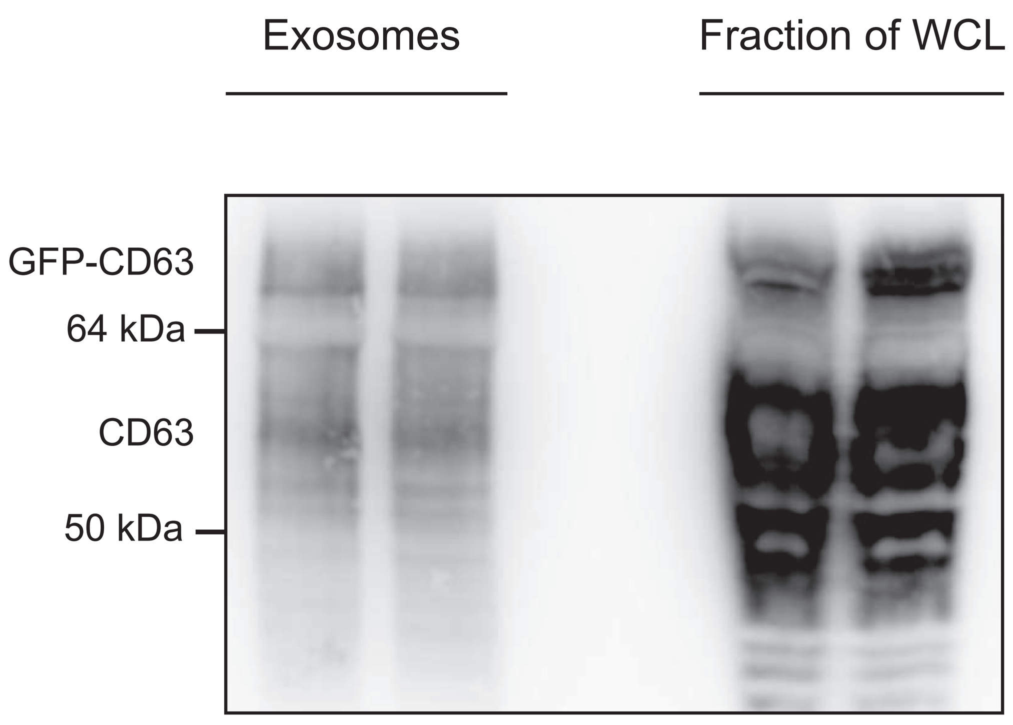

E

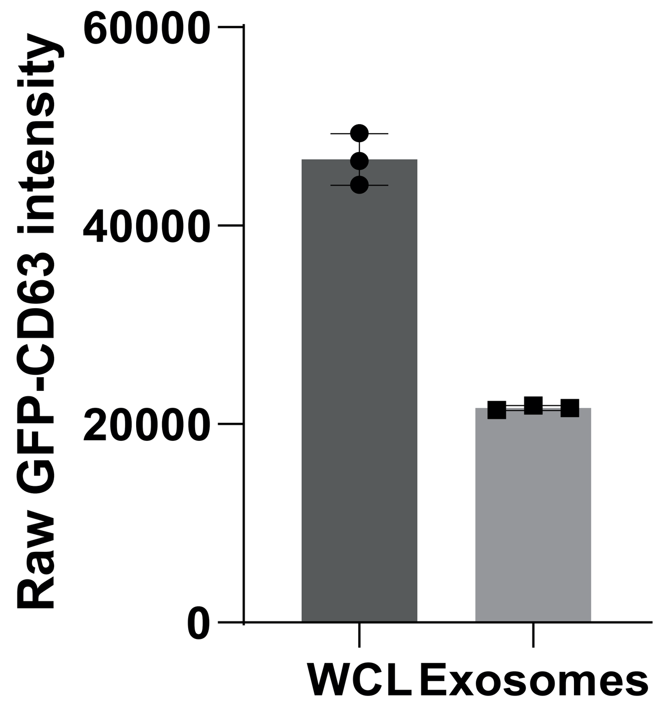

F

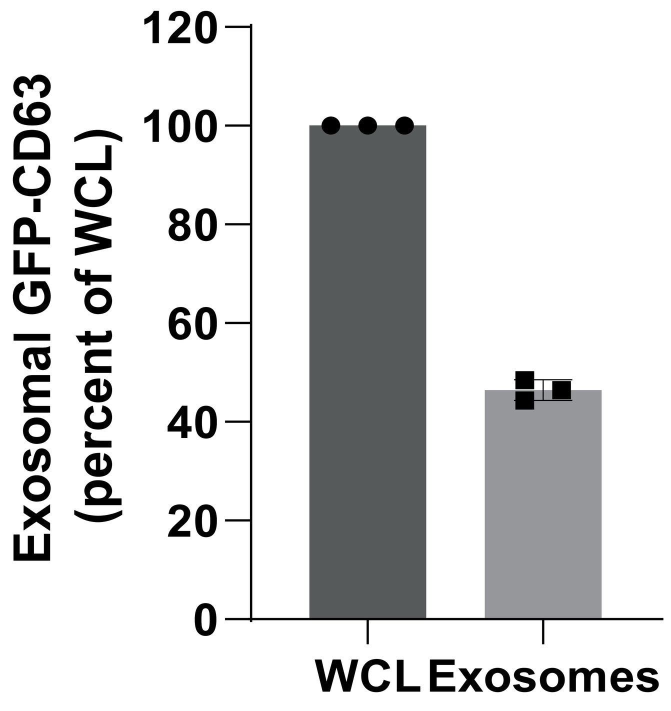

G

**GFP-CD63**	*WCL*	*MVBs*
Degradation + secretion	11%	18%
Degradation	5%	8%
Secretion	6%	10%

H

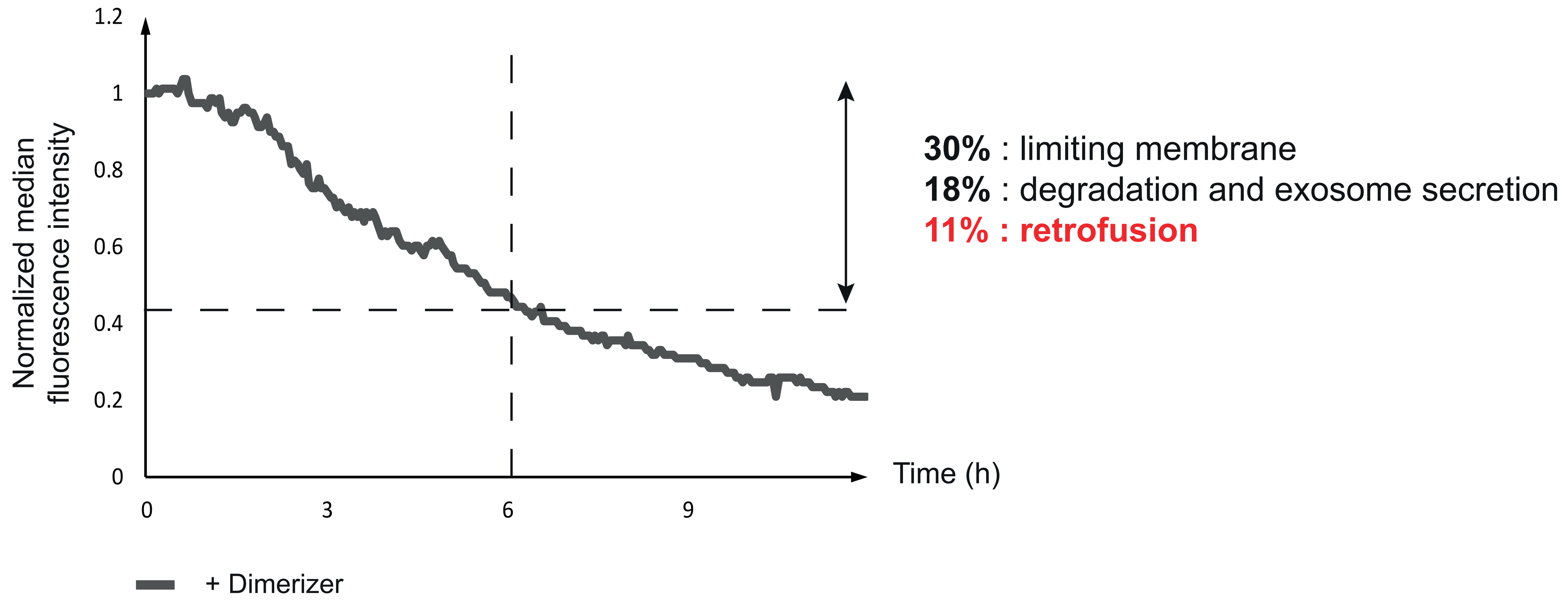

Figure S4. Retrofusion and exosomes. Related to Figure 4.

(A) Electron micrographs of exosome isolates prepared as described in Figure 4F. Scale bar, 200 nm.

(B) Biochemical profile of exosome isolates (secreted in absence of dimerizer) was assessed by immunoblot against the indicated markers. TfR: transferrin receptor, recycling endosomes; HLA-DR: MHC class II, LM and ILVs of late endosomes/lysosomes; Lamp2: LM of late endosomes/lysosomes; Golgin97: trans-Golgi network; Calnexin: endoplasmic reticulum; Tsg101: exosome marker. WCL: diluted whole-cell lysate (to prevent overloading control signal). The position of marker proteins is indicated.

(C) Immunoblot analysis of GFP-CD63 (detected with anti-GFP antibodies) versus untagged CD63 in exosome isolates and a fraction of whole cell lysate (WCL) from control cells or cells overexpressing TQ-IFITM3 treated in the presence (+) or absence (-) of dimerizer. The different incubations as well as the position of the marker proteins are indicated.

(D) Immunoblot analysis of CD63 in exosome isolates and WCL from control cells. Exosomes were isolated from $10x10^6$ cells. For the WCL, $1.3x10^6$ cells were loaded on the same gel. The results from a duplicate exosome isolation were loaded on two lanes as is the corresponding WCL. Position of marker proteins is indicated.

(E) Quantification of GFP-CD63 signal in exosome isolates versus the signal in a fraction of WCL from control cells. Exosomes were isolated from $10x10^6$ cells and $1.3x10^6$ cells were loaded on the same gel. Shown is mean +/- SD from three independent experiments.

(F) Quantification of the percentage of GFP-CD63 secreted in exosomes relative to the fraction cell lysate ($1.3x10^6$ cells) including MVBs. This is not corrected for the number of cells providing exosomes. The corrected values are shown in Figure 4H.
